# Genes associated with MUC5AC expression in small airway epithelium of human smokers and non-smokers

**DOI:** 10.1186/1755-8794-5-21

**Published:** 2012-06-07

**Authors:** Guoqing Wang, Zhibo Xu, Rui Wang, Mohammed Al-Hijji, Jacqueline Salit, Yael Strulovici-Barel, Ann E Tilley, Jason G Mezey, Ronald G Crystal

**Affiliations:** 1 Department of Genetic Medicine, Weill Cornell Medical College, New York, NY, USA; 2 Chengdu Second People’s Hospital, Chengdu, China; 3 Department of Medicine, Division of Pulmonary and Critical Care Medicine, Weill Cornell Medical College, New York, NY, USA; 4 Department of Biological Statistics and Computational Biology, Cornell University, Ithaca, NY, USA; 5 Department of Genetic Medicine, Weill Cornell Medical College, 1300 York Avenue, Box 96, 10065, New York, NY, USA

## Abstract

**Background:**

Mucus hypersecretion contributes to the morbidity and mortality of smoking-related lung diseases, especially chronic obstructive pulmonary disease (COPD), which starts in the small airways. Despite progress in animal studies, the genes and their expression pattern involved in mucus production and secretion in human airway epithelium are not well understood. We hypothesized that comparison of the transcriptomes of the small airway epithelium of individuals that express high *vs* low levels of MUC5AC, the major macromolecular component of airway mucus, could be used as a probe to identify the genes related to human small airway mucus production/secretion.

****Methods**:**

Flexible bronchoscopy and brushing were used to obtain small airway epithelium (10^th^ to 12^th^ order bronchi) from healthy nonsmokers (n=60) and healthy smokers (n=72). Affymetrix HG-U133 plus 2.0 microarrays were used to assess gene expression. Massive parallel sequencing (RNA-Seq) was used to verify gene expression of small airway epithelium from 5 nonsmokers and 6 smokers.

**Results:**

MUC5AC expression varied 31-fold among the healthy nonsmokers. Genome-wide comparison between healthy nonsmokers (n = 60) grouped as “high MUC5AC expressors” *vs* “low MUC5AC expressors” identified 528 genes significantly up-regulated and 15 genes significantly down-regulated in the high *vs* low expressors. This strategy identified both mucus production and secretion related genes under control of a network composed of multiple transcription factors. Based on the literature, genes in the up-regulated list were used to identify a 73 “MUC5AC-associated core gene” list with 9 categories: mucus component; mucus-producing cell differentiation-related transcription factor; mucus-producing cell differentiation-related pathway or mediator; post-translational modification of mucin; vesicle transport; endoplasmic reticulum stress-related; secretory granule-associated; mucus secretion-related regulator and mucus hypersecretory-related ion channel. As a validation cohort, we assessed the MUC5AC-associated core gene list in the small airway epithelium of an independent set of healthy smokers (n = 72). There was up-regulation of MUC5AC in the small airway epithelium of smokers (2.3-fold, p < 10^-8^) associated with a coordinated up-regulation of MUC5AC-associated core gene expression pattern in the small airway epithelium of smokers (p < 0.01). Deep sequencing confirmed these observations.

**Conclusion:**

The identification of the genes associated with increased airway mucin production in humans should be useful in understanding the pathogenesis of airway mucus hypersecretion and identifying therapeutic targets.

**Author summary:**

Mucus hypersecretion contributes to the morbidity and mortality of smoking-related lung diseases, especially chronic obstructive pulmonary disease (COPD), which starts in the small airways. Little is known about the gene networks associated with the synthesis and secretion of mucins in the human small airway epithelium. Taking advantage of the knowledge that MUC5AC is a major mucin secreted by the small airway epithelium, the expression of MUC5AC in small airway epithelium is highly regulated at the transcriptional level and our observation that healthy nonsmokers have variable numbers of MUC5AC^+^ secretory cells in the human small airway epithelium, we compared genome-wide gene expression of the small airway epithelium of high *vs* low MUC5AC expressors from 60 nonsmokers to identify the genes associated with MUC5AC expression. This novel strategy enabled identification of a 73 “MUC5AC-associated core gene” list with 9 categories, which control a series of processes from mucin biosynthesis to mucus secretion. The coordinated gene expression pattern of MUC5AC-associated core genes were corroborated in an independent cohort of 72 healthy smokers. Deep sequencing of small airway epithelium RNA confirmed these observations. This finding will be useful in identifying therapeutic targets to treat small airway mucus hypersecretion.

## Background

The process of mucus production and secretion is central to the normal defense of the lung, with mucus an important component of the airway mucociliary escalator that continuously cleanses the lung of inhaled particulates, pathogens and xenobiotics [1]. Mucus is comprised of several components, but is dominated by the secretory mucins: high molecular weight, disulfide linked, heavily glycosylated proteins [2]. There has been considerable progress in defining the mucin composition of airway mucus in health and disease [3], and regulation of mucin synthesis and secretion in the mouse lung [4-9]. However, little is known about the gene networks associated with the synthesis and secretion of mucins in the human small airway epithelium, the cell population exhibiting the first abnormalities associated with smoking [10-14], and the major site of pathology in chronic obstructive pulmonary disease (COPD) [13,15].

One successful approach to understanding mucin synthesis/secretion in the human lung has been to use information from gene manipulation in murine models to identify genes associated with mucin synthesis/secretion, and to evaluate these genes and their associated genes in samples of human airways or in air-liquid interface cultures of human airway epithelium [3-9,16-22]. In order to expand this knowledge base on a genome wide level, we have developed a novel strategy to define the network of genes linked to MUC5AC expression in the human small airway epithelium. Our approach is based on the understanding that there are no mucus glands in the small airways of humans, and the secretory mucins are produced solely by surface secretory cells [1]. Taking advantage of the knowledge that MUC5AC is a major mucin secreted by the small airway epithelium, the expression of MUC5AC in small airway epithelium is highly regulated at the transcriptional level [17] and our observation that healthy nonsmokers have variable numbers of MUC5AC^+^ secretory cells in the human small airway epithelium, we hypothesized that we could group healthy nonsmokers into small airway epithelium “high” and “low”.

MUC5AC expressors. With this strategy, we compared genome-wide gene expression of the small airway epithelium of high *vs* low MUC5AC expressors to identify the genes associated with MUC5AC expression. Annotating this list of genes with the literature relating to mucus production/secretion enabled identification of a small airway epithelium “MUC5AC-associated core gene” list. As a validation strategy for the MUC5AC-associated core gene list, based on the knowledge that cigarette smoking is an environmental stressor associated with increased mucus production [1-3,17,18,22,23], we assessed MUC5AC and MUC5AC-associated core gene expression in an independent cohort of healthy smokers. Not only was MUC5AC up-regulated in the smoker small airway epithelium, but many of the MUC5AC-associated core genes were as well, confirming the validity of the core gene list as being co-regulated with MUC5AC expression.

## Results

### MUC5AC expression in the small airway epithelium of healthy nonsmokers

Consistent with prior studies of airway mucin gene expression in humans [2,3], only 3 types of mucins (secretion-polymeric, tethered-shed in mucus and tethered) were expressed (P call % >80%) in the small airway epithelium (Table1). Among these 8 mucin genes, we focused on MUC5AC (214385_s_at), a secreted-polymeric mucin, as it is highly expressed by airway surface mucus-producing cells [2,3,18].

**Table 1:** Mucin gene expression in small airway epithelium of healthy nonsmokers

**Category**	**Mucin gene**	**Probeset ID**^**1**^	**%P call**	**Normalized** **expression**^**2**^
Secreted, nonpolymeric	MUC7	217059_at	0	N/A^3^
	MUC8	217295_at	0	N/A
Secreted, polymeric	MUC6	214133_at	8	N/A
	MUC19	1553436_at	0	N/A
	MUC2	204673_at	25	N/A
	MUC5AC	214385_s_at	100	54 ± 44
	MUC5B	213432_at	100	48 ± 29
Tethered, shed in mucus	MUC1	213693_s_at	100	70 ± 18
	MUC16	220196_at	100	46 ± 18
	MUC4	217109_at	100	81 ± 36
Tethered	MUC12	226654_at	6	N/A
	MUC13	218687_s_at	100	5 ± 4
	MUC15	227238_at	100	65 ± 18
	MUC17	232321_at	0	N/A
	MUC20	226622_at	100	46 ± 23
	MUC3A	217117_x_at	50	N/A
	MUC3B	214898_x_at	2	N/A

^1^ If one gene had multiple probe sets, the one with highest % P was used.

^2^ Gene expression data was normalized “per chip” in Genespring 7.0, which is normalized to the median expression level of all the genes on the chip. Data is expressed as mean ± standard deviation.

^3^ N/A, the expression is not shown because % P < 80%.

**Figure 1:**
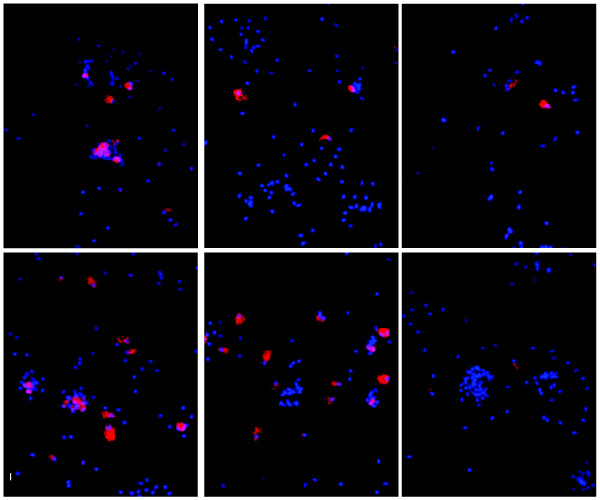
**Variability of proportions of surface MUC5AC**^**+**^**cells in the small airway epithelium of healthy nonsmokers.** Immunofluorescence of MUC5AC staining (red) was processed on cytospin slides prepared from 10^th^-12^th^ order brushed airway epithelial cells. Shown are examples from 6 individuals. Not shown, IgG irrelevant control, negative for MUC5AC staining. Blue - DAPI. Bar = 20 μm.

**Figure 2:**
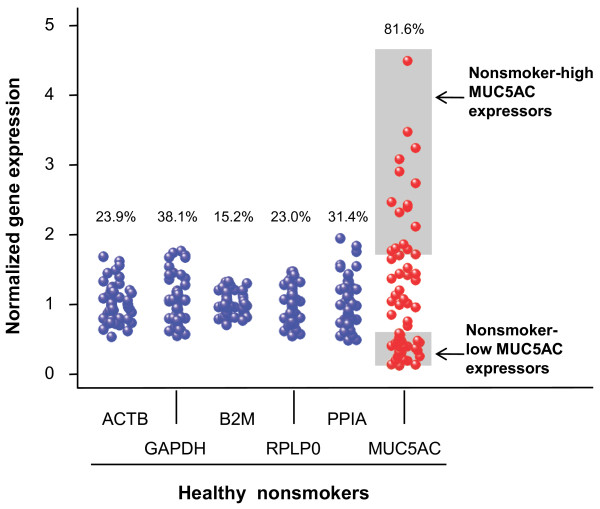
**Healthy nonsmokers were grouped based on MUC5AC gene expression in 10-12**^**th**^**order brushed airway epithelial cells.** Shown is data for n = 60 nonsmokers divided into nonsmoker “high MUC5AC expressors” (MUC5AC expression, highest quartile of all healthy nonsmokers, n = 15, upper shaded region) and nonsmoker “low MUC5AC expressors” (MUC5AC expression, lowest quartile of all healthy non-smokers, n = 15, lower shaded region). Expression of 5 endogenous control genes (ACTB, GAPDH, B2M, RPLP0 and PPIA) in the same samples are used as controls to assess the expression variability in nonsmokers of housekeeping genes compared to MUC5AC. The coefficients of variation (CV) of MUC5AC and endogenous control gene expression are shown.

To assess the proportion of MUC5AC positive cells in the small airway epithelium, MUC5AC staining was carried out on cytospin slides prepared from 10-12th order brushed airway epithelial cells from healthy nonsmokers (Figure1). Consistent with the literature [24,25], there were MUC5AC positive cells in the small airway epithelium of healthy nonsmokers. Importantly, relevant to the present study, there was significant variability from individual to individual in the number of MUC5AC positive cells. In agreement with the histologic findings, healthy nonsmokers showed significant variation in the degree of MUC5AC gene expression. Compared to the housekeeping genes ACTB (CV = 23.9%), GAPDH (CV = 38.1%,), B2M (CV = 15.2%), RPLP0 (CV = 23.0%) and PPIA (CV = 31.4%), the variability of MUC5AC was >2-fold higher (CV = 81.6%; Figure2; Ansari-Bradley test, p < 10^-8^, all comparisons).

### Identification of MUC5AC-associated core genes

**Figure 3:**
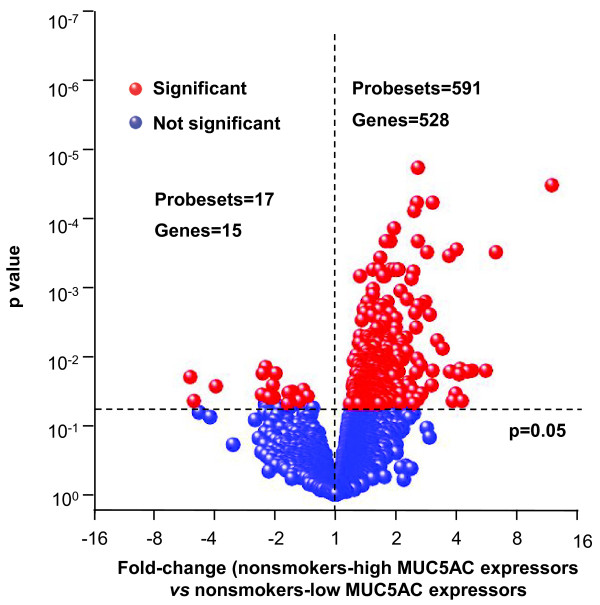
**Identification of MUC5AC-associated core genes.** Shown is a volcano plot comparing the normalized expression of gene probe sets in the 10-12th order brushed airway epithelial cells of nonsmoker-high MUC5AC expressors *vs* low MUC5AC expressors; y-axis - negative log of p value; x-axis - log2-transformed fold-change; red dots - probe sets with significant p value; blue dots - probe sets with non-significant p value. Differentially expressed genes were determined by unequal variance Student’s *t*-test followed by Benjamini-Hochberg correction (p < 0.05). There were 528 genes identified genome-wide that were significantly correlated with MUC5AC high expressors. Of these, 73 genes were identified as MUC5AC-associated core genes based on literature mining (Table1, Additional file 1: Table S1, S2).

In order to identify genes associated with MUC5AC biosynthesis and secretion, we compared the gene expression differences between healthy nonsmoker high MUC5AC expressors *vs* low MUC5AC expressors. To avoid confusion of the dominant transcriptome response to oxidative stress in smokers, only nonsmokers were used to identify genes associated with MUC5AC expression. After controlling false discovery rate by Benjamini-Hochberg correction (p < 0.05), microarray analysis revealed 528 genes were up-regulated and 15 genes were down-regulated in MUC5AC high expressors compared to the MUC5AC low expressors (Figure3; Additional file 1: Table S2).

To explore the function of the up-regulated genes, the online annotation term enrichment tool, DAVID, was used. Genes with functional annotation of Golgi (p < 10^-9^), endoplasmic reticulum (ER; p < 10^-5^), and ER-Golgi transport (p < 2x10^-4^) were enriched in these genes (Additional file 1: Table S3). This is consistent with the knowledge that Golgi, ER apparatus, and transport between ER and Golgi are essential to the secretion pathway [26]. Based on these associations, further literature mining was carried out to identify which genes were known to be associated with or potentially involved in mucus production or secretion.

From 528 up-regulated genes, we found 73 genes with literature supported roles or potential roles in mucus production/secretion. The putative roles of these 73 “MUC5AC-core genes” were grouped into 9 categories: (1) mucus components; (2) mucus-producing cell differentiation-related transcription factors; (3) mucus-producing cell differentiation-related pathways or mediators; (4) post-translational modification of mucin; (5) vesicle transport; (6) endoplasmic reticulum stress-related; (7) secretory granule-associated; (8) mucus secretion-related regulators and (9) mucus hypersecretory-related ion channels (Table2; see Additional file 1: Table S1 for all related references; see Additional file 1: Figure S1, S2 for the likely roles of the products of these genes in airway mucus production and secretion). Within each category, the literature regarding the relevance of each gene to small airway epithelial MUC5AC production and secretion were catalogued as to links in the literature based on non-human models, human cells *in vitro,* human airway epithelium *in vivo* and “functional association”, i.e., likely function in the airway epithelium based on the known function of the gene product.

**Table 2:** **Human small airway epithelium MUC5AC-associated core genes**^**1,2**^

**Mucus components**	**Mucin producing cell differentiation-related**		**Post-translational modification of mucin**	**Vesicle transport**	**Endoplasmic reticulum stress-associated**	**Secretory granule- associated**	**Mucus secretion-related regulators**	**Mucus hypersecretory- related ion channels**
	**Transcription factors**	**Pathways or meditators**						
TFF3	SPDEF	HES1	AGR2	MIA3	CREB3L1	SYTL2	PRSS23	SLC12A2
TFF1	FOXA3	TSTA3	GNE	SURF4	EDEM3	RAB3D	PLCE1	CLCA2
	SOX2	LRRFIP2	GALNT4	KDELR2	XBP1	SCIN	DGKA	SCNN1A
	KLF4	KRAS	GALNT7	KDELR3	EIF2AK3	STXBP6	ITPR3	GABRP
		MAPK13	GALNT12	ITSN1		RAB27B	PRKCD	
		RPS6KA3	PDIA5	ERGIC1		SYTL4		
		CTSC	FUT3	CKAP4		SYTL5		
		SERPINB4	FUT6	GOSR1		GSN		
		PLA2G4A	ST6GAL1	SYNJ2BP		RIMS1		
			ST8SIA1	MPPE1		CASK		
			CHST6	SEC31A		MYO5B		
			GALNT5	ARF4		MYO5C		
			GALNT6	VPS13D		PCLO		
				SEC22B		PAM		
				TPD52		ATP6V0A4 KIF5B CDC42EP5		

^1^ Genes with a role in mucus production or mucus secretion (based on the literature) were selected from the significantly up-regulated genes in nonsmoker high-MUC5AC expressors compared to nonsmoker low-MUC5AC expressors (see Supplemental Methods for complete criteria).

^2^ References for the functions of each gene are in Additional file 1: Table S1. See Additional file 1: Figure S1, S2 for the schematic illustration of the functions of these genes relevant to MUC5AC-production/secretion.

From the assignments based on the literature, 27 of the 73 MUC5AC-core genes have been previously identified as being associated with mucus production or secretion, including those associated with mucus components (TFF3, TFF1); mucus-producing cell differentiation related-transcription factors (SPDEF, FOXA3, SOX2 and KLF4); mucus-producing cell differentiation-related pathways or mediators (HES1 and TSTA3, Notch pathway; RPS6KA3 and KRAS, MAPK pathway; PLA2G4A; CTSC; SERPINB4); post-translational modification of mucin (AGR2, GNE, GALNT4, GALNT7, GALNT12); secretory granule-associated (SYTL2, RAB27B, RAB3D, SCIN, MYO5B, MYO5C); mucus secretion-related regulators (ITPR3, PRKCD); and a mucus hypersecretory-related ion channel (SLC12A2). Of these 27 MUC5AC-core genes known to be strongly linked to mucus production and secretion, most have been linked to airway mucus production and secretion by murine or *in vitro* studies; only 9 have been previously linked to MUC5AC production/secretion in human *in vivo* studies (Additional file 1: Table S1).

Of the remaining 46 MUC5AC-core genes, although they have not been strongly linked to airway mucus production/secretion, there is literature supporting their roles in mucus production/secretion in other cell types, including mucus-producing cell differentiation-related pathways or mediators (Wnt pathway gene, LRRFIP2 and MAPK pathway gene, MAPK13); post-translational modification of mucin (FUT3, FUT6, ST6GAL1, ST8SIA1, CHST6, GALNT5, GALNT6); vesicle transport (MIA3, SURF4, KDELR2, KDELR3, ITSN1, ERGIC1, CKAP4, GOSR1, SYNJ2BP, MPPE1, SEC31A, ARF4, VPS13D, SEC22B and TPD52); ER stress-related (EIF2AK3, EDEM3, XBP1 and CREB3L1); secretory granule-associated (RIMS, PCLO, PAM, ATP6V0A4, KIF5B, CDC42EP5); mucus secretion-related regulators (PRSS23, DGKA, PLCE1); and mucus hypersecretory-related ion channels (CLCA2, SCNN1B and GABAP) (Table2, Additional file 1: Table S1).

Together, the MUC5AC-core genes suggest that multiple molecular events involving the nucleus, endoplasmic reticulum, Golgi, vesicles and plasma membrane work coordinately in the human small airway epithelium to promote mucus production and secretion in MUC5AC-producing cells.

### MUC5AC-core genes in smokers

**Figure 4:**
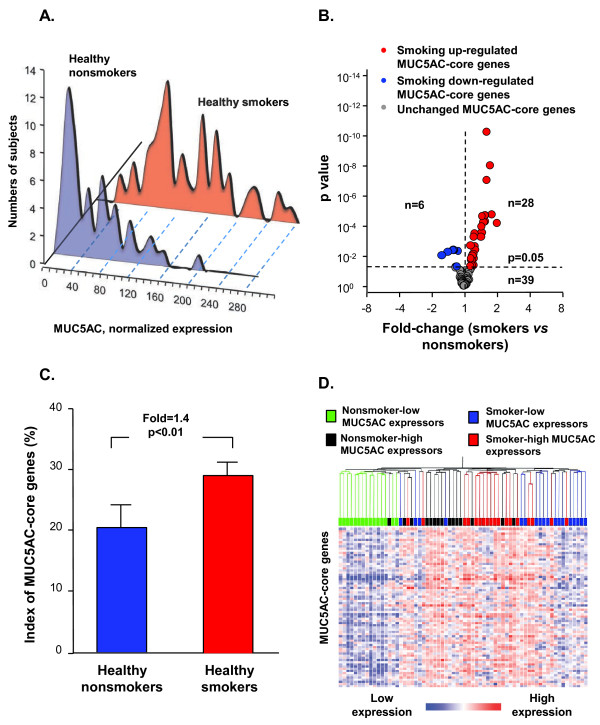
**cMUC5AC expression and MUC5AC-core genes in the small airway epithelium of healthy smokers. A.** MUC5AC gene expression distribution in healthy smokers (blue, n = 60) compared to healthy nonsmokers (red, n = 72). Y-axis, number of subjects; X-axis, normalized MUC5AC expression. **B.** Volcano plot of MUC5AC-core genes comparing healthy smokers and healthy nonsmokers. Differentially expressed genes between smokers (n = 72) and nonsmokers (n = 60) were determined by unequal variance Student’s *t*-test followed by Benjamini-Hochberg correction (p < 0.05). Only MUC5AC-core genes were plotted. y-axis - negative log of p value; x-axis - log2-transformed fold-change; red dots – smoking up-regulated MUC5AC-core genes; blue dots – smoking down-regulated MUC5AC-core genes; grey dots – smoking unchanged MUC5AC-core genes. Numbers of each group are shown. **C.** Index of expression of the MUC5AC-core genes (% of abnormally expressed MUC5AC-core genes beyond that of healthy nonsmoker MUC5AC low-expressors in healthy nonsmokers (blue) compared to healthy smokers (red). Fold-change represents the average index difference between the groups, p value indicates significant differences between the groups. **D.** Unsupervised hierarchical clustering analysis of expression of MUC5AC-core genes in nonsmoker-high MUC5AC expressors (black, highest quartile of all healthy non-smokers, n = 15), nonsmoker-low MUC5AC expressors (green, lowest quartile of all healthy non-smokers, n = 15), smoker-high MUC5AC expressors (red, highest quartile of all healthy smokers, n = 18) and smoker-low MUC5AC expressors (blue, lowest quartile of all healthy smokers, n = 18). Genes expressed above the average are represented in red, below average in blue, and average in white.

Chronic cigarette smoking, with its 4000 xenobiotics and >10^14^ oxidants per puff, is a complicated stress to the airway epithelium of smokers [22,27]. Based on this, and the knowledge that smoking induces metaplasia of MUC5AC positive cells in the airway epithelium [1-3,22], and prior literature detailing increased expression of MUC5AC in the airway epithelium of smokers [3,28,29], we used small airway epithelium of chronic smokers as a validation set, i.e., if the human MUC5AC-core genes identified in the nonsmoker small airway epithelium are truly associated with MUC5AC synthesis/secretion, then the MUC5AC-core genes should be up-regulated in the small airway epithelium of smokers. As expected, healthy smokers had 2.3-fold higher MUC5AC gene expression than nonsmokers (Figure4A). Like the nonsmokers, there was considerable variability in MUC5AC expression among smokers [MUC5AC coefficient of variation among smokers 47.1%, compared to 25.3 ± 3.1% for the 5 housekeeping genes, ACTB, GAPDH, B2M, RPLP0 and PPIA (Ansari-Bradley test, all p < 10^-11^ for comparison between MUC5AC and each housekeeping gene)]. Comparison of the expression patterns of MUC5AC-core genes between healthy smokers and healthy nonsmokers demonstrated that 28 of the 73 (38%) MUC5AC-core genes were up-regulated in smokers (Figure4B Additional file 1: Table S4). Interestingly, 6 out of the 73 MUC5AC-core genes were down-regulated by smoking, suggesting smoking has a variable effect on some components of the orchestrated MUC5AC-production/secretion system in the small airway epithelium.

To assess these global smoking-related modifications of the MUC5AC core genes, we created an overall index of the MUC5AC-core genes. The index analysis supported the concept that healthy smokers had more active MUC5AC-core gene expression (up- or down-regulation) compared to the nonsmokers (p < 0.01, Figure4C). Clustering analysis comparing the high and low MUC5AC expression in nonsmokers and smokers globally demonstrated that there were 2 major groups among smokers and nonsmokers, based on expression of the MUC5AC-core genes, with nonsmoker-high MUC5AC expressors having a similar expression pattern of MUC5AC-core genes to smoker-high MUC5AC expressors (Figure4D). Together, these results suggest that most MUC5AC-core genes are shared by smokers and nonsmokers in association with high MUC5AC expression.

**Figure 5:**
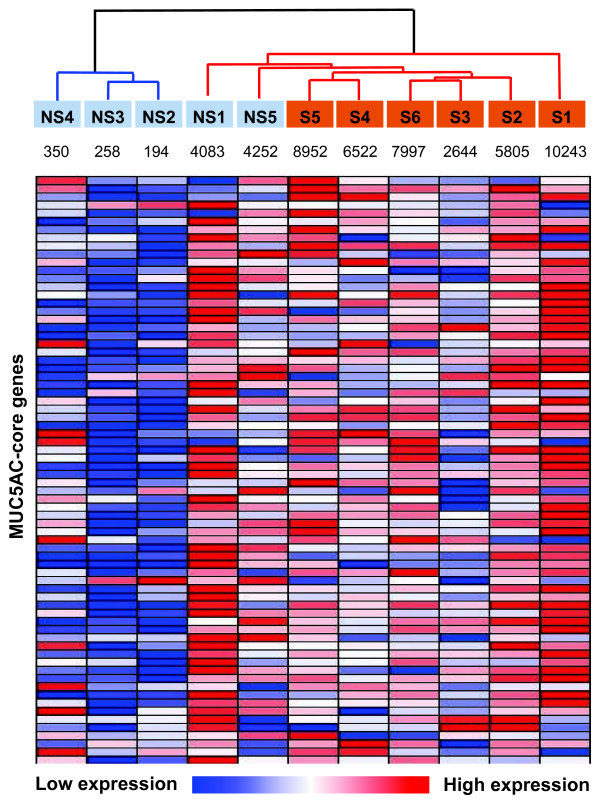
**MUC5AC-core genes assessed by RNA-Seq analysis in healthy smokers and healthy nonsmokers.** Shown is a hierarchical cluster analysis of RNA-Seq data of expression of the MUC5AC-associated core genes from 5 healthy nonsmokers and 6 healthy smokers. Smoking status was represented by different colors (nonsmoker, blue; smoker, brown). In the heat map, gene expression above the average is represented in red, below average in blue, and average in white. The color schemes represent the relative expression level across each row (each gene). Dendrogram color denotes there are two different groups independent of smoking status. The expression level (RPKM) of MUC5AC is shown under the label of each individual (NS = nonsmoker; S = smoker).

To confirm the microarray findings, RNA sequencing was used to assess the gene expression profile from airway epithelium of 5 nonsmokers and 6 smokers. Consistent with microarray data, RNA-Seq data showed that MUC5AC-core genes had a coordinated expression pattern (Figure5). As with the microarray data, in general, smokers had more active MUC5AC-core gene expression, although similar to the microarray data, there was overlap, with some nonsmokers having high levels of MUC5AC-core gene expression.

### MUC5AC-core genes in asthma

Asthma is known to be associated with airway mucus overproduction. To assess the effect of asthma on MUC5AC-core gene expression, the data set from a study by Woodruff et al. [30] was used. Comparison of the expression patterns of MUC5AC-core genes between asthma patients at baseline and healthy nonsmokers demonstrated that 6 of the 73 (8%) MUC5AC-core genes were up-regulated and 1 MUC5AC-core gene was down-regulated in asthma (Additional file 1: Table S6). To assess whether expression of MUC5AC-core genes were correlated with MUC5AC in this asthma study, we performed genome-wide correlation analysis to MUC5AC gene expression. Interestingly, 10 of 73 MUC5AC-core genes are among the top 300 genes correlated with MUC5AC (p < 10^-4^Additional file 1: Table S7).

### Gene networks linking the MUC5AC-core genes

**Figure 6:**
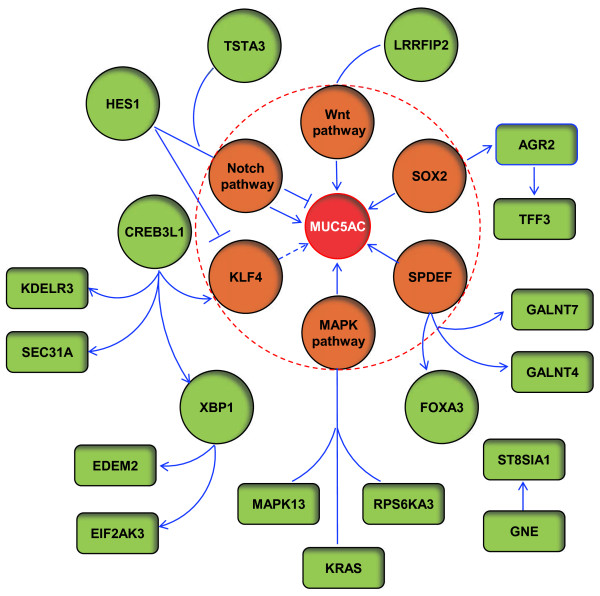
**Proposed gene network of the MUC5AC-core genes.** From the MUC5AC-core gene list, genes with at least one literature supported connection to other MUC5AC-core genes were selected (see Methods for detail). The connection denotes that the expression level of one gene is affected by the other gene; the data is derived from literature with *in vitro* gene overexpression/activation/knockdown experiments or transgenic mice data (see Additional file 1: Table S5 for references). Protein to protein interactions are not included. Genes in the brown solid circle include, transcription factors (SPDEF, SOX2, KLF4) and the Wnt, Notch, MAPK pathways, which can affect MUC5AC-producing cell differentiation. A red dashed circle was used to emphasize their important roles. Genes in green solid circle represent transcription factors in the MUC5AC-core gene list with literature supported potential role in MUC5AC-producing cells. Genes in green rectangles represent non-transcription factors in the MUC5AC-core gene list. Arrows indicate upstream gene can regulate downstream gene expression. Dashed arrow, based on mouse data [31], KLF4 can affect mucus-producing cell differentiation, but there is no direct evidence showing KLF4 can affect MUC5AC expression. The solid line linked to pathway indicates that MAPK13, KRAS and RPS6KA3 belong to “MAPK pathway”; HES1 and TSTA3 belong to “Notch pathway”; LRRFIP2 belong to “Wnt pathway”. The “T” like symbol indicates a potential blocking effect.

To help understand the underlying mechanism of the coordinate expression of the MUC5AC-core genes, a gene network was built with the focus on transcriptional control. Based on the knowledge from murine studies or *in vitro* studies that inhibition of the transcription factors Spdef, Sox2 and Klf4 or perturbation of Wnt, Notch and MAPK pathway affects differentiation of mucus-producing cells or expression of MUC5AC [4-9,31], these genes were used as the backbone of the network (Figure6). We manually screened each of 73 human small airway MUC5AC-core genes for their transcriptional connections with other MUC5AC-core genes. Only those genes with connections with other MUC5AC-core genes were integrated into the network. These known interactions among MUC5AC-core genes suggest that the findings based on the comparison between high and low MUC5AC expressors were not just by chance. The network was centered on MUC5AC, but, interestingly, involved multiple transcription factors and regulatory pathways. Downstream genes of these transcription factors or pathways are linked to different steps of mucus production, which imply the coordinated expression patterns of MUC5AC-core genes are orchestrated by these transcription factors or pathways. Notably, SPDEF and CREB3L1 seem to be two major “driving forces” in this network. The gene network also provides clues to understand the function of other MUC5AC-associated genes in mucus production.

The EGF receptor family related signaling pathway plays an import role in mucus production [32]. However, our approach to identify MUC5AC-core genes did not identify EGF receptor family genes as MUC5AC-associated genes. Interestingly, overexpression of EGF receptor 2 (ERBB2, HER2) has been demonstrated in primary lung mucinous adenocarcinomas, which were characterized by KRAS activating mutations as well as very high levels of MUC5AC production [33,34]. To investigate the possible relation between ERBB2 and MUC5AC at the transcriptome level, we performed genome-wide correlation analysis for ERBB2 in both healthy nonsmokers and healthy smokers. Although EGF receptor-associated pathways were enriched in ERBB2 correlated genes (Additional file 1: Table S8), no close relation between ERBB2 and mucus production-related genes were found.

## Discussion

Mucus hypersecretion is a cardinal feature of chronic airway diseases and mucins are the major component of airway mucus [1-4,17,18,23]. Despite the significant progress in understanding the transcriptional control, biosynthesis and secretion of mucins using murine models, advances in understanding the coordinated gene expression relevant to producing and secreting mucins in the human airway have been limited due to the lack of methods to isolate and culture pure populations of primary human airway mucin-producing cells. Taking advantage of the knowledge that MUC5AC is the major secreted mucin of the small airway epithelium, and our observation that there is variable small airway epithelium MUC5AC expression among healthy nonsmokers, we hypothesized that genome-wide comparison of small airway gene expression of healthy individuals with “high” MUC5AC expression to those with “low” MUC5AC expression would identify genes whose regulation paralleled that of MUC5AC.

From a list of 528 genes up-regulated in healthy high MUC5AC expressors compared to healthy low MUC5AC expressors, we identified 73 MUC5AC core genes that had literature support for a role in mucin production/secretion, from murine studies of the airways, immunohistochemical or *in situ* hybridization studies of human airway epithelium, studies from mucin-secreting cell lines, or studies relating to mucin production/secretion in organs other than lung. These genes included those categorized as mucus components, mucus-producing cell differentiation-related transcription factors, mucus-producing cell differentiation-related pathways or mediators, post-translational modification of mucin, vesicle transport, ER stress-associated, secretory granule-associated, mucus secretion-related regulators and mucus hypersecretory related ion channels. While some of these 73 human small airway epithelium core MUC5AC genes have been previously associated with human airway MUC5AC expression, for most, this is the first demonstration that, in the human airways, these genes are coordinately up-regulated along with MUC5AC expression. As an independent validation set, we assessed the expression of these 73 core MUC5AC genes in the small airway epithelium of healthy smokers, where there is a stress on the airway epithelium associated with up-regulation of MUC5AC expression [1-4,17,18,22,23]. The data demonstrates that, as in the “high” *vs* “low” healthy nonsmokers, the healthy smokers up-regulate 38% of the MUC5AC-core genes. Finally, and consistent with studies in knockout and transgenic mice, MUC5AC expression in the human small airway epithelium was linked to several mucus production/secretion-related transcription factors (SOX2, KLF4, SPDEF, XBP1 and CREB3L1), as well as the Notch, Wnt and MAPK pathways, that, in turn, control coordinated expression of many of the MUC5AC-core genes identified in the present study. These findings expand the knowledge regarding molecular events underlying human airway MUC5AC-related production/secretion and provide several potential targets for medical intervention for mucus hypersecretion.

### MUC5AC expression in the human small airway epithelium

MUC5AC is one of the major secretory mucins expressed by surface airway epithelial cells, and represents a marker for the airway surface mucus-producing cells [1-4,17,18,22,23]. As demonstrated in the present study and in several other studies [25,28,29], there is up-regulation of MUC5AC expression in the airway epithelium of smokers compared to nonsmokers. This observation is consistent with the knowledge that, on the average, smoking is associated with increased airway mucus production [1-4,17,18,22,23]. However, novel to the present study is the observation that MUC5AC expression in the small airway epithelium, assessed at both the mRNA and protein levels, is highly variable among healthy nonsmokers, implying that there are some individuals more prone to airway mucus production than others. We capitalized on this observation to categorize healthy nonsmokers into “high” and “low” MUC5AC expressors. Then, using genome wide assessment of gene expression, we compared “high” *vs* “low” MUC5AC expressors to identify genes, the expression of which correlates with MUC5AC expression. Using the literature to identify genes already known to be associated with the airway epithelium (mostly from murine studies), as well as to identify genes associated with mucin expression in other organs, we were able to define “MUC5AC-core” genes i.e., the genes plausibly linked to MUC5AC gene expression in the human small airway epithelium. To test the validity of the MUC5AC-core genes, we hypothesized that many of these genes would be up-regulated further in the small airway epithelium of smokers, since smokers have, on the average, up-regulation of MUC5AC. This proved to be correct.

### Mucus-producing cell differentiation-related transcription factors, pathways and mediators

Studies of MUC5AC expression in experimental non-human models have identified several transcription factors that modulate mucus-producing cell differentiation, including Stat6, Spdef, Klf4, Sox2 and Foxa2 [4,5,20,31,35]. In addition to these transcription factors, murine studies have identified several signaling pathways, including Wnt [6], Notch [7,8] and MAPK [9] pathways, which have been implicated in differentiation of MUC5AC-producing cells.

Consistent with these observations, the MUC5AC-core genes linked to MUC5AC expression in the human small airway epithelium in the present study include the mucus-producing cell differentiation-related transcription factors, SPDEF, KLF4, SOX2 and FOXA3. Up-regulation of FOXA3 and SPDEF have been reported during mucus overproduction in human airways cells *in vitro* and *in vivo*[4]. SPDEF can also drive differentiation of MUC5AC-producing cells in primary human airway epithelial cells *in vitro*[4]*.* The demonstration that SOX2 and KLF4 are linked to MUC5AC in the human airway epithelium is novel to the present study. The MUC5AC-core genes also included the Wnt pathway gene LRRFIP2, Notch pathway genes HES1 and TSTA3 [19], and the MAPK pathways genes MAPK13, KRAS [36], and RPS6KA3 (RSK2) [37] as linked to MUC5AC expression in the human small airway epithelium. Together, this suggests that a gene network, instead of single gene or pathway, controls differentiation of MUC5AC-producting cells.

The EGF receptor family mediated signaling pathway is involved in mucus production [32]. One of the EGF receptor family members, ERBB2, has been shown to be overexpressed in lung mucinous adenocarcinomas [33,34]. However, no correlation between ERBB2 and MUC5AC at transcription level was found in the current study using normal airway epithelium. These results suggest that MUC5AC expression is not dependent on the expression level of EGF receptors in the normal airway epithelium. Instead, activation status of EGF receptors might be more important, while in mucinous adenocarcinomas, the constitutive activation of the ERBB2 pathway, which is caused by persistent up-regulation of ERBB2 and ERBB2 mutations, might contribute to the MUC5AC overexpression.

In addition to these transcription factors and pathways, it is known that several enzyme-related genes can mediate mucus-producing cell differentiation [38-40]. Consistent with these observations, we found that SERPINB4 (serpin peptidase inhibitor), CTSC (peptidase) and PLA2G4A (phospholipase) are up-regulated in high MUC5AC expressors. Serpinb3a (SERPINB4 homolog) deletion markedly attenuates mucus-producing cell hyperplasia in mouse airway after allergen challenge [38]; ozone and Staphylococcal enterotoxin B exposure is associated with CTSC-driven mucus production in the mouse [39]; and PLA2G4A activation is associated with mucus overproduction in murine airway [40]. Both SERPINB4 and CTSC are up-regulated in IL13 treated human primary bronchial epithelial cells on air liquid interface culture [16], and PLA2G4A is more active in bronchial explants from individuals with cystic fibrosis [40]. It is a novel observation that these genes are linked to small airway MUC5AC production/secretion in healthy humans.

### MUC5AC biosynthesis

MUC5AC is a polymeric mucin which is heavily glycosylated and thus the ER and Golgi apparatus play a central role in biosynthesis of MUC5AC. To form the mature MUC5AC protein, a stepwise posttranslational modification is needed, including N-glycosylation, dimer formation, O-glycosylation, fucose/sialic acid/sulfate modification and polymer formation, processes involving several specific enzymes [2,3]. Among these, several were identified among the MUC5AC core genes.

Consistent with the findings from mouse or human *in vitro* studies, we found that AGR2 (protein disulfide isomerases) [41], GALNT4 and GALNT7 (GalNAc-T members) [4] and GNE (required for normal sialylation) [42] were up-regulated in high-MUC5AC expressors. Besides these genes, we found some novel enzyme genes that are likely involved in human MUC5AC posttranslational modifications, including PDIA5 (disulfide isomerase), GALNT5, GALNT6 and GALNT12 (GalNAc-T members), FUT3 and FUT6 (fucosyltransferase), ST6GAL1 and ST8SIA1 (sialic acid transferase). Interestingly, PDIA5 is also up-regulated in Barrett’s esophagus, a disorder in which mucus-producing cells are a basic pathologic change [43].

Along the secretory pathway, vesicle-mediated transportation is important to carry newly synthesized proteins from the ER to the Golgi [17]. To maintain the function of the ER, reverse transportation is also active, which balances the loss of protein embedded in the vesicles from the ER [44]. Consistent with this concept, we found 15 vesicle transport related genes associated with high MUC5AC expression, including MIA3, SURF4, KDELR2, KDELR3, ITSN1, ERGIC1, CKAP4, GOSR1, SYNJ2BP, MPPE1, SEC31A, ARF4, VPS13D, SEC22B, and TPD52. None have been associated with mucus production in the literature.

Mucin biosynthesis places a large metabolic burden on the cell, with potential ER and Golgi apparatus stress [45]. In agreement with the concept that mucus deregulation might induce ER stress, EIF2AK3 and XBP1 (classical unfolded protein response genes), EDEM3 (endoplasmic reticulum-associated degradation gene), and CREB3L1 (non-canonic unfolded protein response gene) were up-regulated in high-MUC5AC expressors. EIF2AK3 can repress global protein synthesis [46], XBP1 can induce chaperone synthesis [47], and EDEM3 can promote the degradation of poor-quality proteins [48]. Finally, the Drosophila ortholog of CREB3L1, CrebA, has been shown to be the major and direct regulator of secretory capacity in the salivary gland [49]. Our findings raise the possibility that the human airway surface MUC5AC-producing cells likely use an ER stress*-*associated signal as feedback to finely control mucus production.

### MUC5AC secretion

MUC5AC secretion is highly regulated [17]. As shown in the “secretory granule-associated” and “mucus secretion-related regulator” categories, activation of the mechanisms underlying mucin secretion was reflected in the function of MUC5AC-core genes. For example, we found PRSS23, PLCE, PRKCD, DKGA, ITPR3, RAB3D, RAB27B, MYO5B, MYO5C, SYTL2, SYTL4, SYTL5, SCIN, CDC42EP5, ATP6V0A4, STXBP6, PAM, RIMS1, PLCO and CASK were up-regulated in high-MUC5AC expressors. PRSS23 is a serine protease, which can stimulate mucus glycoprotein release from hamster tracheal ring organ culture [50]. PLCE is a PLC family member that might be involved in generating the intracellular second messengers, IP3 and DAG, in mucus-producing cells [1]. ITPR3 is the receptor of IP3 [1]. PRKCD is a protein kinase C family member. RAB3D and RAB27B belong to RAB3 and RAB27 family, respectively [17]. ATP6V0A4 and PAM are associated with maturation of secretion granules [51]. Both MYO5B and MYO5C are type V myosins, and SYTL2 (SLP2A) [52], SYTL4 and SYTL5 are granulophilins [17]. KIF5B is associated with granule movement [53]. SCIN (scinderin) is an enzyme that is needed for actin disruption [17] and gelsolin can affect actin remodeling [54]. RIMS1 (RIM), PCLO (Piccolo), and CASK can help secretory granule tethering and docking [17]. STXBP6 belongs to Munc18 family. Among this group of MUC5AC-core genes, SYTL4, SYTL5, RIMS1, PCLO, CASK, KIF5B, CDC42EP5, ATP6V0A4, STXBP6, PAM, DKGA, and PLCE have not been previously identified in association with mucus-producing cells.

### Mucus hypersecretion-related ion channels

Several studies have suggested that ion channels are involved in mucus production, including CFTR [55], SLC26A4 (pendrin) [56], SCNN1B (ENaC subunit) [21], CLCA family [57], GABAergic system receptors [58] and SLC12A2 [59]. In the current study, we found SCNN1A (ENaC alpha subunit), CLCA2 (CLCA family member), GABAP (a subunit of the GABAergic system receptor), SLC12A2 (Slc12a2 homolog) were up-regulated in high MUC5AC expressors. Among them, SLC12A2 expression is known to be restricted to human airway mucus-producing cells [59]. The links between SCNN1A, CLCA2, GABAP and MUC5AC expression in human airway are novel. Since multiple ion channel genes are found to be associated with MUC5AC expression, these observations suggest an active water/ion exchange in the airway epithelial cell membrane during human airway mucus production.

Several limitations of this study should be pointed out. First, all MUC5AC-core genes were identified based on the literature, and thus, important genes might be ignored because we are lacking clues from current knowledge. Second, our strategy was based on the transcriptome level, so regulation mechanisms depending on protein level modulation are underestimated.

## Methods

### Nomenclature

The terminology associated with mucus-producing cells in the airway epithelium varies in the literature, including the terms “goblet cells” and “mucous cells” [1,23]. Since we are focusing on genes correlating with the expression of MUC5AC, to avoid confusion, we use the term “MUC5AC-producing cells” as the surface airway epithelium cells (the cells that are sampled with bronchoscopy and brushing) expressing MUC5AC. When referring to the literature, if the mucus-related gene/protein is not clear, the term “mucus-producing cells” will be used.

### Ethics statement

All individuals were evaluated at the Weill Cornell NIH Clinical and Translational Science Center and Department of Genetic Medicine Clinical Research Facility, using Institutional Review Board-approved clinical protocols.

### Study population

Healthy nonsmokers and healthy smokers were recruited from the general population in New York City. The nonsmokers were used as the “test” set to identify the MUC5AC-core genes; the smokers were used as a “validation” set to determine if the MUC5AC-core genes correlate with MUC5AC gene expression under conditions (smoking) where MUC5AC gene expression is known to be up-regulated.

The criteria for “healthy” was based on a history, physical exam, complete blood count, coagulation studies, liver function tests, urine studies, chest X-ray, EKG and pulmonary function tests (Table3; see Supplemental Methods for inclusion/exclusion criteria). All subjects were negative for HIV1 and had normal α1-antitrypsin levels. Urine nicotine and cotinine levels were measured using liquid chromatography-tandem mass spectrometry (ARUP laboratories, Salt Lake City, UT). “Nonsmokers” (n = 60) were defined as self-reported life-long nonsmokers, with non-detectable urine nicotine (<2 ng/ml) and cotinine (<5 ng/ml); “Smokers” (n = 72) were defined as self-reported current smokers with urine nicotine >2 ng/ml and/or urine cotinine >5 ng/ml. No individuals smoked within 12 hr prior to the bronchoscopy procedure to collect the airway epithelium.

**Table 3:** **Study population and biologic samples**^**1**^

**Parameter**	**Healthy nonsmokers**	**Healthy smokers**
n	60	72
Gender (male/female)	38/22	51/21
Age	41 ± 12	43 ± 8
Race (B/W/O)^2^	27/23/10	45/16/11
Smoking history (pack-yr)	0	27 ± 16
Urine nicotine (ng/ml)	negative	1098 ± 1312
Urine cotinine (ng/ml)	negative	1228 ± 939
Pulmonary function parameters^4^		
FVC (% predicted)	106 ± 13	110 ± 14
FEV1 (% predicted)	106 ± 14	110 ± 15
FEV1/FVC (% observed)	82 ± 6	81 ± 4
TLC (% predicted)	99 ± 13	100 ± 12
DLCO (% predicted)	98 ± 14	94 ± 11
Epithelial cells^4^		
Number recovered x10^6^	6.5 ± 2.9	7.4 ± 3.3
% epithelial cells^5^	99.1 ± 1.2	99.0 ± 1.3
% inflammatory cells	0.9 ± 1.2	1.0 ± 1.3
Differential cell count^6^		
Ciliated (%)	72.0 ± 8.9	64.0 ± 12
Secretory (%)	6.7 ± 3.7	8.4 ± 4.4
Basal (%)	12.6 ± 6.6	14.5 ± 8.0
Intermediate (%)	7.8 ± 3.4	12.2 ± 6.8

^1^ Data are presented as mean ± standard deviation.

^2^ B = Black, W = White, O = Other.

^3^ Pulmonary function testing parameters are given as % of predicted value with the exception of FEV1/FVC, which is reported as % observed; FVC - forced vital capacity; FEV1 - forced expiratory volume in 1 sec; TLC - total lung capacity; DLCO - diffusing capacity.

^4^ To quantify the percentage of epithelial and inflammatory cells and the proportions of ciliated, basal, secretory, and intermediate epithelial cells, aliquots of 2x10^4^ cells were prepared by centrifugation and stained with DiffQuik.

^5^ Of total cells recovered.

^6^ Of total epithelial cells recovered.

### Epithelial sampling, cDNA preparation and microarray processing

Airway epithelium (10^th^ to12^th^ order) was collected by fiberoptic bronchoscopy by brushing as previously described [14]. The expression of genes encoding surfactant and Clara cell secretory proteins confirmed the samples were derived from the small airway epithelium [14]. Cytopreparations were prepared by centrifugation (Cytospin 11, Shandon Instruments, Pittsburgh, PA) and stained with Diff-Quik (Dade Behring, Newark, NJ). For MUC5AC staining in small airway epithelium, cytospin slides of brushed cells were stained with mouse anti-human MUC5AC antibody (Vector, Burlingame, CA) and detected by Cy3 labeled goat anti-mouse antibody (Jackson, West Grove, PA). Nuclei were counterstained with DAPI (Invitrogen, Carlsbad, CA).

RNA in the airway epithelium samples was processed for microarray analysis as previously described [14,60]. Total RNA was extracted from the small airway epithelium using a modification of the TRIzol method (Invitrogen, Carlsbad, CA) and processed to generate cDNA. Genome-wide gene expression analysis was performed using HG-U133 Plus 2.0 array (Affymetrix, Santa Clara, CA) according to Affymetrix protocols, hardware and software. Overall microarray quality was verified by the criteria: (1) 3'/5' ratio for GAPDH ≤3; and (2) scaling factor ≤10.0 [61]. The captured image data from the HG-U133 Plus 2.0 arrays was processed using the MAS5 algorithm. The raw data are available at the Gene Expression Omnibus (GEO) site (http://www.ncbi.nlm.nih.gov/geo/); accession number for this dataset is GSE34450.

### Mucin gene expression in the small airway epithelium

The mucin genes represented on the HG-U133 Plus 2.0 array were grouped as: secretion-nonpolymeric; secretion-polymeric; tethered-shed in mucus; and tethered [2,3]. For genes with multiple probe sets, the one with highest expression level in nonsmokers was chosen. The percentage of Affymetrix “P call” was calculated for each probeset. To compare expression levels among mucin genes, Genespring data normalized “per chip” (normalized to the median of all the genes on the same chip) was used.

To evaluate the expression variability of MUC5AC, endogenous controls were used as references. Five endogenous control genes were randomly selected from literature (ACTB, GAPDH, B2M, RPLP0 and PPIA) [62] with “per chip” normalized gene expression level >99% genes (probesets) on the HG-U133 Plus 2.0 array. Coefficient of variation (CV) of expression of MUC5AC and the control genes were calculated. For control genes with multiple probesets, the one with the highest CV was chosen. Genespring “per chip” and “per gene” (normalized to the median of the same gene across all samples) normalizations were used. Comparison of the variance of MUC5AC *vs* control gene expression (“per chip” and “per gene” normalized data) was done using the Ansari-Bradley test in R language (http://www.r-project.org/).

### Identification of MUC5AC-associated core genes

To identify the MUC5AC-associated core genes, MUC5AC expression of the healthy nonsmokers was quantified using probeset 214385_s_at, the probeset generating the strongest signal among the 3 of MUC5AC probesets on the microarray. Healthy nonsmokers were categorized as high MUC5AC expressors (MUC5AC expression highest quartile, n = 15), medium MUC5AC expressors (the 2^nd^ and 3^rd^ quartiles; n = 15 and n = 15, respectively) and low MUC5AC expressors (MUC5AC expression lowest quartile, n = 15). Genome-wide analysis of the genes associated with high and low MUC5AC expressors were filtered by the criteria: (1) Affymetrix “P call” >80% in nonsmokers; and (2) genes with known annotations. Significant differences of gene expression between high MUC5AC expressors and low MUC5AC expressors were determined by an unequal variance Student’s *t*-test followed by Benjamini-Hochberg correction (B-H correction, p < 0.05). The probe sets of MUC5AC were not included. For genes represented by more than one probeset, the probeset with the lowest p value was used. The Database for Annotation, Visualization and Integrated Discovery (DAVID) was used to do gene function annotation term enrichment analysis of the significant up-regulated genes [63].The role of each up-regulated gene in mucus production and secretion was assessed by manual literature mining. Genes with literature supporting roles in mucus production or secretion were defined as the “MUC5AC-core genes” (see Supplemental methods for complete criteria).

### Effects of smoking on expression of MUC5AC and MUC5AC-core genes

As a validation cohort to assess the role of smoking on MUC5AC expression and the MUC5AC-core genes, MUC5AC expression (probeset 214385_s_at) was compared in the small airway epithelium of healthy nonsmokers (n = 60) and healthy smokers (n = 72). The comparison of variability of MUC5AC to the 5 housekeeping genes in smokers was analyzed with the Ansari-Bradley test. To determine if the MUC5AC-core genes identified in nonsmokers were also up-regulated in smokers, genome-wide transcriptome comparison between healthy smokers (n = 72) and healthy nonsmokers (n = 60) was based on the filtered probesets (using same filter criteria used to identify the MUC5AC-associated core genes). Significant differences of gene expression between smokers and nonsmokers were determined by an unequal variance Student’s *t*-test followed by Benjamini-Hochberg correction (p <0.05). The results of MUC5AC-core genes (fold-change and Benjamini-Hochberg corrected p value) were then extracted.

Cluster analysis was performed on microarray data (“per chip” and “per gene” normalization) of the MUC5AC-core genes in nonsmokers and smokers using settings with Spearman correlation as similarity measure and the average linkage clustering algorithm. To reduce the variability associated with medium MUC5AC expressors, only nonsmoker-high MUC5AC expressors (highest quartile of all healthy nonsmokers, n = 15), nonsmoker-low MUC5AC expressors (lowest quartile of all healthy nonsmokers, n = 15), smoker-high MUC5AC expressors (highest quartile of all healthy smokers, n = 18) and smoker-low MUC5AC expressors (lowest quartile of all healthy smokers, n = 18) were used.

### Asthma study

GEO4302 datasets [30] were used to evaluate the effect of asthma on MUC5AC-core genes, using data from 42 asthmatics and 28 healthy subjects in this data set. Microarray data were processed by RMA algorithm in Genespring 7.0. Significant differences of gene expression between asthma and healthy subjects were determined by an unequal variance Student’s *t*-test followed by Benjamini-Hochberg correction (p < 0.05). Genome-wide correlations to MUC5AC (214385_s_at) were assessed by Spearman’s rank correlation coefficient.

### MUC5AC-core gene index

A “MUC5AC-core gene index” was created as a global measure of the coordinate MUC5AC-enhanced expression-associated changes by integrating information on expression levels of all MUC5AC-core genes using the conceptual framework described by Tilley et al. [64]. For each gene, the mean and standard deviation were calculated from the values in nonsmoker-low MUC5AC expressors. The “normal range” was defined as within 2 standard deviations (SD) of the mean. The MUC5AC-core gene index for each nonsmoker and smoker was defined as a percentage of MUC5AC-core genes with expression levels outside the normal range, using the formula:

(1) $$\text{MUC5AC}(\%)=\sum_{n=1}^{n}cEn$$

where E1 has a value of 1 if the expression level for gene 1 was >2 SD beyond that of nonsmoker-low MUC5AC expressors or a value of 0 if the expression level was ≤2 SD below that of nonsmoker-low MUC5AC expressors; E2 is the index for gene 2, etc., and the constant c [c = 100 divided by the number of MUC5AC-core genes (*n*) normalizes the index to the percent of MUC5AC-core genes that are outside of the range of healthy nonsmoker-low expressors]. The statistical significance of differences in index between the groups was determined using the Mann Whitney *U*-test.

### RNA-seq data analysis

Analysis of the MUC5AC-core genes was also carried out using a database of massive parallel sequencing (RNA-Seq) of the transcriptome of healthy nonsmokers (n = 5) and healthy smokers (n = 6) [65]. Because there is no MUC5AC sequence in the reference genome build UCSC hg19, the resultant reads were aligned to *Homo sapiens* high coverage assembly GRCh37 using Bowtie v 0.12. Reads per kilobase of exon model per million mapped reads (RPKM) was used to quantify transcript levels of each gene. The sequence read data have been submitted to the NCBI SRA database (SRA accession #SRP005411). Of the 73 MUC5AC-core genes, 72 were used for clustering analysis (GALNT4 does not have an ensembl sequence). Log2 transformed gene level RNA-Seq data was used for hierarchical clustering analysis, which was performed in “HierarchicalClustering” of Genepattern (http://genepattern.broadinstitute.org) with the default setting (Distance measure: Pearson correlation; Clustering method: pairwise complete-linkage). To visualize the heatmap, “HierarchicalClusteringViewer” of Genepattern was used with the default setting (View Options are “relative color schemes” and “gradient color”).

### Gene network analysis

A gene network analysis was built on the human small airway epithelium MUC5AC-core genes by manually data mining the literature. The network focused on transcription control, using transcription factors/pathways known to be associated with mucin biosynthesis (SPDEF, SOX2 and KLF4 and Wnt, Notch and MAPK pathways) as the network backbone. MUC5AC-core genes with transcriptional/pathway level connections with other MUC5AC-core genes were selected to expand the network. The interconnections among genes were based on literature with gene overexpression/activation/knockdown experiments *in vitro* or data from genetically engineered mice showing that the expression level of one gene is affected by another gene. Protein-protein interactions were not included (See Additional file 1: Table S5 for details).

## Competing interests

The authors have no competing interests.

## Authors’ contributions

GW, ZX, RW and MAH conceptualized and designed the study. YSB, AET and RGC oversaw patient recruitment, sample acquisition and experimental protocols. JGM contributed to the design of the analytic strategy. JS and GW performed the statistical and computational analyses. GW and RW interpreted the results, and wrote the manuscript. RGC supervised the analyses and edited the manuscript. All authors read and approved the final manuscript.

## Pre-publication history

The pre-publication history for this paper can be accessed here:

http://www.biomedcentral.com/1755-8794/5/21/prepub

## Supplementary Material

Additional file 1 **Inclusion and Exclusion Criteria for Healthy Nonsmokers and Healthy Smokers**[1,2,4-6,8,9,16,17,19,21,30,31][36-43,46-54,57-59,66-76]. Click here for file
