# Supplementary material for: Genes associated with MUC5AC expression in small airway epithelium of human smokers and non-smokers
Source: BMC Med Genomics. 2012 Jun 7;5:21. doi: 10.1186/1755-8794-5-21 (PMC3443416; doi:10.1186/1755-8794-5-21)
Supplement: Additional file 1 — Inclusion and Exclusion Criteria for Healthy Nonsmokers and Healthy Smokers[1,2,4-6,8,9,16,17,19,21,30,31][36-43,46-54,57-59,66-76]. [file 1755-8794-5-21-S1.pdf]

Supplemental Methods

Inclusion and Exclusion Criteria for Healthy Nonsmokers and Healthy Smokers

Healthy nonsmokers

Inclusion criteria

- Males and females, at least 18 years old
- Provide informed consent
- Good health without history of chronic lung disease, including asthma, and without recurrent or recent (within 3 months) acute pulmonary disease
- Normal physical examination
- Normal routine laboratory evaluation, including general hematologic studies, general serologic/immunologic studies, general biochemical analyses, and urine analysis
- Normal α1-antitrypsin level
- HIV negative
- Normal PA and lateral chest X-ray
- Normal electrocardiogram (sinus bradycardia, premature atrial contractions are permissible)
- Not pregnant (females)
- No history of allergies to medications used in the bronchoscopy procedure
- Not taking any medications relevant to lung disease or having an effect on the airway epithelium
- Willingness to participate in the study
- Self-reported nonsmokers, with smoking status validated by the absence of nicotine and cotinine in urine

Exclusion criteria

- Unable to meet the inclusion criteria
- Current active infection or acute illness of any kind
- Alcohol or drug abuse within the past 6 months
- Evidence of malignancy within the past 5 years

Healthy smokers

Inclusion criteria

- Males and females, at least 18 years old
- Provide informed consent
- Good health without history of chronic lung disease, including asthma, and without recurrent or recent (within 3 months) acute pulmonary disease
- Normal physical examination
- Normal routine laboratory evaluation, including general hematologic studies, general serologic/immunologic studies, general biochemical analyses, and urine analysis
- Normal α1-antitrypsin level
- HIV negative
- Normal PA and lateral chest X-ray
- Normal electrocardiogram (sinus bradycardia, premature atrial contractions are permissible)

- Not pregnant (females)
- No history of allergies to medications used in the bronchoscopy procedure
- Not taking any medications relevant to lung disease or having an effect on the airway epithelium
- Willingness to participate in the study
- Self-reported current daily smokers with any number of pack-yr, validated by urine nicotine >1000 ng/ml and/or cotinine >1000 ng/ml

Exclusion criteria

- Unable to meet the inclusion criteria
- Current active infection or acute illness of any kind
- Alcohol or drug abuse within the past 6 months
- Evidence of malignancy within the past 5 years

Criteria Used to Identify MUC5AC-associated Core Genes

From all up-regulated genes in high-MUC5AC expressors compared to low-MUC5AC expressors, genes involved or potentially involved in mucus production/secretion were identified based on the literature. These literature supported genes were identified as the MUC5AC-associated core genes. The putative roles of mucus production / secretion related genes were grouped into the following 9 categories and genes from the up-regulated 528 genes were selected if they could be assigned to these categories based on a survey of the literature. The brief functional descriptions of these selected genes are provided in Supplemental Table I. The rationale for these categories are as follows.

1. Mucus components (rationale: some mucus components are co-secreted with MUC5AC from the same cells)
2. Mucus-producing cell differentiation-related transcription factors (rationale: transcription factors can control mucus-producing cell differentiation)
3. Mucus-producing cell differentiation-related pathways or mediators (rationale: several pathways and mediator are involved in mucus-producing cell differentiation)
4. Post-translational modification of mucin (rationale: disulfide bond formation, adding sialic acid, sulfate, fucose and glycosylation are needed for mature MUC5AC protein formation)
5. Vesicle transport (rationale: vesicle mediated protein transportations between ER and Golgi are needed for mucin production)
6. Endoplasmic reticulum stress-related (rationale: active mucus production will cause a heavy burden to ER-Golgi apparatus, which might cause endoplasmic reticulum stress)
7. Secretory granule-associated (rationale: muc5ac forms secretory granules)
8. Mucus secretion-related regulators (rationale: after stimulation, mucin secretion is regulated)
9. Mucus hypersecretory-related ion channels (rationale: mucus secretion and ion/water balance have a close-relationship)

Supplemental Table I. Identification and Categorization of 73 MUC5AC Core Genes[1]

Gene symbol	Gene name	MUC5AC high expressors compared to MUC5AC low expressors (fold-change)[2]	p value[3]	Literature documenting link to mucus production/secretion[4]			Known function makes it likely associated with mucus production/secretion[8]	Summary of function related to mucus production/secretion	Reference
				Non-human models[5]	*In vitro*[6]	*In vivo*[7]			
**Mucus components**									
TFF3	trefoil factor 3 (intestinal)	3.8	$4x10^{-4}$	Y	Y	Y	-	Secreted by airway mucus-producing cells	[1]
TFF1	trefoil factor 1	4.1	$3x10^{-4}$	Y	Y	Y	-	Secreted by mucus-producing cells	[2]
**Mucin producing cell differentiation related transcription factors**									
SPDEF	SAM pointed domain containing ets transcription factor	2.1	$2x10^{-2}$	Y	Y	Y	-	Key factor for airway goblet cell differentiation in mouse	[3]
FOXA3	forkhead box A3	2.6	$6x10^{-5}$	Y	Y	Y	-	Strong staining in goblet cells	[3]
SOX2	SRY (sex determining region Y)-box 2	1.4	$1x10^{-2}$	Y	N	N	-	Affects goblet cell differentiation in murine airway	[4]
KLF4	Kruppel-like factor 4 (gut)	2.1	$6x10^{-4}$	Y	N	N	-	Affects goblet cell differentiation in murine colon	[5]
**Mucus-producing cell differentiation-related pathways or mediators**									
HES1	hairy and enhancer of split 1, (Drosophila)	1.7	$4x10^{-2}$	N	Y	N	-	Belongs to NOTCH pathway; can affect secretory cell differentiation in murine small intestine and colon	[6]
TSTA3	tissue specific transplantation antigen P35B	1.4	$2x10^{-2}$	Y	N	N	-	Belongs to NOTCH pathway; can affect secretory cell differentiation in murine small intestine and colon	[7]
LRRFIP2	leucine rich repeat (in FLII) interacting protein 2	1.3	$7x10^{-4}$	N	N	N	Y	Belongs to WNT pathway; can activate beta catenin	[8]
KRAS	v-Ki-ras2 Kirsten rat sarcoma viral oncogene homolog	1.3	$3x10^{-2}$	Y	N	N	-	Belongs to MPAK pathway involved in mucus production; also involved in EGFR pathway	[9]
MAPK13	mitogen-activated protein kinase 13	1.5	$6x10^{-3}$	N	N	N	Y	Belongs to MPAK pathway involved in mucus production	[10]
RPS6KA3	ribosomal protein S6 kinase, 90kDa, polypeptide 3	1.4	$4x10^{-2}$	N	Y	N	-	Alias , RSK2; belongs to MPAK pathway involved in mucus production	[11]
CTSC	cathepsin C	1.7	$1x10^{-2}$	Y	N	N	-	Mediates ozone and Staphylococcal enterotoxin B exposure associated mucus production	[12]
SERPINB4	serpin peptidase inhibitor, clade B (ovalbumin), member 4	3.3	$6x10^{-3}$	Y	Y	Y	-	Mucus-producing cell hyperplasia markedly attenuated in the murine homolog Serpinb3a-null mice after allergen challenge	[13]
PLA2G4A	phospholipase A2, group IVA (cytosolic, calcium-dependent)	1.7	$3x10^{-2}$	Y	Y	Y	-	Alias, cPLA2α; activation associated with mucus overproduction	[14]

Supplemental Table I. Identification and Categorization of 73 MUC5AC Core Genes[1] (cont., page 2)

Gene symbol	Gene name	MUC5AC high expressors compared to MUC5AC low expressors (fold-change)[2]	p value[3]	Literature documenting link to mucus production/secretion[4] Non-human models[5]	*In vitro*[6]	*In vivo*[7]	Known function makes it likely associated with mucus production/secretion[8]	Summary of function related to mucus production/secretion	Reference
**Post-translational modification of mucins**									
AGR2	anterior gradient homolog 2 (Xenopus laevis)	2.6	$2x10^{-4}$	Y	Y	Y	-	Disulfide isomerase for mucin.	[15]
GNE	glucosamine (UDP-N-acetyl)-2-epimerase/N-acetylmannosamine kinase	2.0	$2x10^{-4}$	N	Y	N	-	Required for normal sialylation; up-regulated by IL13-induced goblet cell metaplasia	[16]
GALNT4	UDP-N-acetyl-alpha-D-galactosamine:polypeptide N-acetylgalactosaminyltransferase 4 (GalNAc-T4)	1.7	$3x10^{-3}$	Y	Y	N	-	Involved in mucin chain glycosylation	[3]
GALNT7	UDP-N-acetyl-alpha-D-galactosamine:polypeptide N-acetylgalactosaminyltransferase 7 (GalNAc-T7)	1.8	$2x10^{-3}$	Y	N	N	-	Involved in mucin chain glycosylation	[3]
GALNT12	UDP-N-acetyl-alpha-D-galactosamine:polypeptide N-acetylgalactosaminyltransferase 12 (GalNAc-T12)	1.5	$6x10^{-3}$	Y	N	N	-	Family member affects mucin chain glycosylation	[17]
PDIA5	protein disulfide isomerase family A, member 5	1.7	$6x10^{-4}$	N	N	N	Y	Disulfide isomerase	[18]
FUT3	fucosyltransferase 3 (galactoside 3(4)-L-fucosyltransferase, Lewis blood group)	2.1	$1x10^{-3}$	N	N	N	Y	Fucosyltransferase, likely catalyzes fucose transfer to mucin	[17,19]
FUT6	fucosyltransferase 6 (alpha (1,3) fucosyltransferase)	1.6	$2x10^{-2}$	N	N	N	Y	Fucosyltransferase, catalyzes fucose transfer	[17]
ST6GAL1	ST6 beta-galactosamide alpha-2,6-sialyltranferase 1	1.6	$3x10^{-2}$	N	N	N	Y	Glycosyltransferase, catalyzes sialic acid transfer	[17]
ST8SIA1	ST8 alpha-N-acetyl-neuraminide alpha-2,8-sialyltransferase 1	1.9	$6x10^{-3}$	N	N	N	Y	Glycosyltransferase, catalyzes sialic acid transfer	[17]
CHST6	carbohydrate (N-acetylglucosamine 6-O) sulfotransferase 6	1.6	$3x10^{-2}$	N	N	N	Y	Catalyzes sulfate transfer	[17]
GALNT5	UDP-N-acetyl-alpha-D-galactosamine:polypeptide N-acetylgalactosaminyltransferase 5 (GalNAc-T5)	2.1	$6x10^{-4}$	N	N	N	Y	Family member likely affects mucin chain glycosylation	[17]

Supplemental Table I. Identification and Categorization of 73 MUC5AC Core Genes[1] (cont., page 3)

Gene symbol	Gene name	MUC5AC high expressors compared to MUC5AC low expressors (fold-change)[2]	p value[3]	Literature documenting link to mucus production/secretion[4]			Known function makes it likely associated with mucus production/secretion[8]	Summary of function related to mucus production/secretion	Reference
				Non-human models[5]	*In vitro*[6]	*In vivo*[7]			
GALNT6	UDP-N-acetyl-alpha-D-galactosamine:polypeptide N-acetylgalactosaminyltransferase 6 (GalNAc-T6)	2.4	$8x10^{-4}$	N	N	N	Y	Family member affects mucin chain glycosylation	[17]
**Vesicle transport**									
MIA3	melanoma inhibitory activity family, member 3	1.5	$6x10^{-4}$	N	N	N	Y	COPII vesicle coat subunits Sec23/Sec24 associated	[20]
SURF4	surfeit 4	1.6	$4x10^{-2}$	N	N	N	Y	Maintains the architecture of the endoplasmic reticulum-Golgi intermediate compartment and of the Golgi	[20]
KDELR2	KDEL (Lys-Asp-Glu-Leu) endoplasmic reticulum protein retention receptor 2	1.5	$1x10^{-2}$	N	N	N	Y	Retention of luminal endoplasmic reticulum proteins	[20,21]
KDELR3	KDEL (Lys-Asp-Glu-Leu) endoplasmic reticulum protein retention receptor 3	2.5	$8x10^{-5}$	N	N	N	Y	Retention of luminal endoplasmic reticulum proteins	[20,21]
ITSN1	intersectin 1 (SH3 domain protein)	1.5	$2x10^{-2}$	N	N	N	Y	May regulate formation of clathrin-coated vesicles	[20]
ERGIC1	endoplasmic reticulum-golgi intermediate compartment (ERGIC) 1	1.7	$4x10^{-2}$	N	N	N	Y	Endoplasmic reticulum-Golgi intermediate compartment	[20]
CKAP4	cytoskeleton-associated protein 4	1.8	$7x10^{-3}$	N	N	N	Y	Protein transport between ER and Golgi	[20]
GOSR1	golgi SNAP receptor complex member 1	1.3	$1x10^{-2}$	N	N	N	Y	Involved in transport from the ER to the Golgi apparatus as well as in intra-Golgi transport	[20]
SYNJ2BP	synaptojanin 2 binding protein	1.6	$4x10^{-2}$	N	N	N	Y	Regulates membrane traffic	[20]
MPPE1	Metallophosphoesterase 1	1.7	$1x10^{-2}$	N	N	N	Y	Protein transport between ER and Golgi	[20]
SEC31A	SEC31 homolog A (S. cerevisiae)	1.3	$7x10^{-3}$	N	N	N	Y	Component of the coat protein complex II	[20]
ARF4	ADP-ribosylation factor 4	1.3	$1x10^{-2}$	N	N	N	Y	May modulate vesicle budding	[20]
VPS13D	vacuolar protein sorting 13 homolog D (S. cerevisiae)	1.5	$2x10^{-2}$	N	N	N	Y	Involved in trafficking of membrane proteins between the trans-Golgi network	[20]
SEC22B	SEC22 vesicle trafficking protein homolog B (S. cerevisiae)	1.3	$3x10^{-2}$	N	N	N	Y	Plays a role in the ER-Golgi protein trafficking	[20]
TPD52	tumor protein D52	1.5	$2x10^{-3}$	N	N	N	Y	Potentially involved in vesicle trafficking	[20]

Supplemental Table I. Identification and Categorization of 73 MUC5AC Core Genes[1] (cont., page 4)

Gene symbol	Gene name	MUC5AC high expressors compared to MUC5AC low expressors (fold-change)[2]	p value[3]	Literature documenting link to mucus production/secretion[4]			Known function makes it likely associated with mucus production/secretion[8]	Summary of function related to mucus production/secretion	Reference
				Non-human models[5]	*In vitro*[6]	*In vivo*[7]			
**Endoplasmic reticulum stress-associated reticulum stress-associated**									
CREB3L1	cAMP responsive element binding protein 3-like 1	2.6	$2x10^{-5}$	N	N	N	Y	Orthologue CrebA affects multiple secretory pathway way genes in the secretory tissues of the Drosophila embryo	[21]
EDEM3	ER degradation enhancer, mannosidase alpha-like 3	1.6	$4x10^{-3}$	N	N	N	Y	Involved in ERAD (endoplasmic reticulum associated degradation) for glycoproteins	[22]
XBP1	X-box binding protein 1	1.4	$5x10^{-2}$	Y	N	N	Y	Promote secretory machinery in plasma cells	[23]
EIF2AK3	eukaryotic translation initiation factor 2-alpha kinase 3	1.6	$1x10^{-3}$	N	N	N	Y	Alias, PERK; represses translational initiation in response to ER stress in plasma cells	[24]
**Secretory granule-associated**									
SYTL2	synaptotagmin-like 2	1.6	$4x10^{-3}$	Y	N	N	-	Alias, SLP2A; is a granulophilin, modulates exocytosis of mucin granules and secretion	[25]
RAB3D	RAB3D, member RAS oncogene family	1.6	$2x10^{-2}$	Y	N	N	-	Involved in regulated mucin granule exocytosis	[26]
SCIN	scinderin	1.5	$4x10^{-2}$	Y	Y	N	-	Affect the microfilament network during mucin granule exocytosis	[27]
STXBP6	syntaxin binding protein 6 (amisyn)	1.6	$5x10^{-2}$	N	N	N	Y	Alias, AMISYN; modulates the formation of functional SNARE complexes (essential for granule secretion)	[28]
RAB27B	RAB27B, member RAS oncogene family	1.6	$2x10^{-2}$	Y	N	N	-	Family member RAB27A is important for mucin granule maturation, docking, priming	[26]
SYTL4	synaptotagmin-like 4	1.7	$2x10^{-2}$	N	N	N	Y	Modulates exocytosis of dense-core granules and secretion; family member SYTL2 (Slp2a) affects mucin granule maturation	[26]
SYTL5	synaptotagmin-like 5	1.6	$2x10^{-2}$	N	N	N	Y	Modulates exocytosis of dense-core granules and secretion; family member SYTL2 (Slp2a) affects mucin granule maturation	[26]
GSN	gelsolin (amyloidosis, Finnish type)	2.1	$1x10^{-2}$	N	N	N	Y	Affect actin disruption during exocytosis	[29]
RIMS1	regulating synaptic membrane exocytosis 1	1.6	$3x10^{-2}$	N	N	N	Y	Alias, RIM; is a Rab effector (involved in granule exocytosis)	[26]
CASK	calcium/calmodulin-dependent serine protein kinase (MAGUK family)	1.3	$1x10^{-2}$	N	N	N	Y	Involved in granule docking	[26]
MYO5B	myosin VB	1.4	$4x10^{-2}$	Y	N	N	-	Class V myosin; might be involved in mucin granule transport	[26]
MYO5C	myosin VC	1.3	$3x10^{-2}$	Y	N	N	-	Class V myosin; might be involved in mucin granule transport	[26]
PCLO	piccolo (presynaptic cytomatrix protein)	1.4	$2x10^{-2}$	N	N	N	Y	Alias, Piccolo; likely has a role in synaptic vesicle trafficking and involved in granule docking	[26]

Supplemental Table I. Identification and Categorization of 73 MUC5AC Core Genes[1] (cont., page 5)

Gene symbol	Gene name	MUC5AC high expressors compared to MUC5AC low expressors (fold-change)[2]	p value[3]	Literature documenting link to mucus production/secretion[4]: Non-human models[5]	*In vitro*[6]	*In vivo*[7]	Known function makes it likely associated with mucus production/secretion[8]	Summary of function related to mucus production/secretion	Reference
PAM	peptidylglycine alpha-aminating monooxygenase	1.8	$9x10^{-3}$	N	N	N	Y	Involved in secretory granule maturation	[30]
ATP6V0A4	ATPase, H+ transporting, lysosomal V0 subunit a4	1.6	$3x10^{-2}$	N	N	N	Y	Belongs to vacuolar proton ATPase family, involved in secretory granule maturation	[30]
KIF5B	kinesin family member 5B	1.3	$3x10^{-2}$	N	N	N	Y	Alias, Kinesin-1, involved in granule movement	[31]
CDC42EP5	CDC42 effector protein (Rho GTPase binding) 5	3.1	$6x10^{-5}$	N	Y	N	Y	Downstream gene of CDC42, which is essential for mucin granule secretion	[26,32]
**Mucus secretion-related regulators**									
PRSS23	protease, serine, 23	1.8	$3x10^{-3}$	N	N	N	Y	Serine proteases stimulate mucus glycoprotein release	[33]
DGKA	diacylglycerol kinase, alpha 80kDa	1.7	$2x10^{-3}$	N	N	N	Y	Diacylglycerol is messenger of mucus secretion signal	[26,34]
PRKCD	protein kinase C, delta	1.5	$2x10^{-2}$	N	Y	N	-	Protein kinase C family has important roles in mucus secretion	[26,34]
ITPR3	inositol 1,4,5-triphosphate receptor, type 3	1.6	$5x10^{-3}$	Y	N	N	-	IP3 receptor is a cellular messenger of mucin secretion signals	[26,34]
PLCE1	phospholipase C, epsilon 1	1.5	$3x10^{-2}$	N	N	N	Y	PLC family plays a central role in regulated mucin secretion	[26,34]
**Mucus hypersecretory related ion channels**									
SLC12A2	solute carrier family 12 (sodium/potassium/chloride transporters), member 2	1.6	$2x10^{-2}$	Y	N	Y	-	Highly expressed in mucus-producing cells during mucus hypersecretion	[35]
CLCA2	chloride channel accessory 2	2.6	$4x10^{-3}$	N	N	N	Y	Family member CLCA1 is involved in mucus hypersecretion	[36]
SCNN1A	sodium channel, non voltage-gated 1 alpha	1.4	$3x10^{-2}$	N	N	N	Y	Alias, ENaCalpha; overexpression causes goblet cell metaplasia in mice	[37]
GABRP	gamma-aminobutyric acid (GABA) A receptor, pi	2.2	$1x10^{-2}$	N	N	N	Y	GABAergic system is involved in mucus hypersecretion in murine airway	[38]

1 Literature mining identified genes with a role in mucus production or mucus secretion (see supplemental text for complete criteria).
2 Fold-change, nonsmoker-high MUC5AC expressors compared to nonsmoker-low MUC5AC expressors.
3 p value, nonsmoker-high MUC5AC expressors compared to nonsmoker-low MUC5AC expressors. Significant differences of gene expression between high MUC5AC expressors and low MUC5AC expressors were determined by an unequal variances Student's t test followed by Benjamini-Hochberg correction ($p <0.05$).
4 Listed are Y=yes or N=no if MUC5AC-core genes fulfill or do not fulfill, respectively, the criteria shown in the column header; –, non-applicable.
5 Previously linked to mucus production/secretion in nonhuman models (*in vitro*/*in vivo*, airway epithelial or non airway epithelial cells).
6 Previously linked to mucus production/secretion in human cells *in vitro* (airway epithelial or non-epithelial cells, cell lines/primary cells).
7 Previously linked to mucus production/secretion in human airway epithelium *in vivo*.
8 Not previously linked to mucus production /secretion (footnotes 3-5), but included in the MUC5AC-associated core gene list because it is correlated with MUC5AC high expression and known function makes it likely associated with mucus production/secretion.

Supplemental Table II. Genes Differently Expressed Between Nonsmoker-high MUC5AC Expressors Compared to Nonsmoker-low MUC5AC Expressors

Gene symbol[1]	Gene name	Fold-change[2]	p value[3]
**Up-regulated**			
**CREB3L1**	cAMP responsive element binding protein 3-like 1	2.6	$2x10^{-5}$
**CEACAM5**	carcinoembryonic antigen-related cell adhesion molecule 5	12.6	$4x10^{-5}$
**CDC42EP5**	CDC42 effector protein (Rho GTPase binding) 5	3.1	$6x10^{-5}$
**FOXA3**	forkhead box A3	2.6	$6x10^{-5}$
**KDELR3**	KDEL (Lys-Asp-Glu-Leu) endoplasmic reticulum protein retention receptor 3	2.5	$8x10^{-5}$
**GNE**	glucosamine (UDP-N-acetyl)-2-epimerase/N-acetylmannosamine kinase	2.0	$2x10^{-4}$
**AGR2**	anterior gradient homolog 2 (Xenopus laevis)	2.6	$2x10^{-4}$
BCL2L14	BCL2-like 14 (apoptosis facilitator)	1.9	$2x10^{-4}$
TCEA3	transcription elongation factor A (SII), 3	1.8	$2x10^{-4}$
**TFF1**	trefoil factor 1	4.1	$3x10^{-4}$
FAM83D	family with sequence similarity 83, member D	2.9	$3x10^{-4}$
**TFF3**	trefoil factor 3 (intestinal)	3.8	$4x10^{-4}$
NIPAL2	NIPA-like domain containing 2	1.7	$4x10^{-4}$
GALE	UDP-galactose-4-epimerase	1.9	$6x10^{-4}$
**GALNT5**	UDP-N-acetyl-alpha-D-galactosamine:polypeptide N-acetylgalactosaminyltransferase 5 (GalNAc-T5)	2.1	$6x10^{-4}$
KCNE3	potassium voltage-gated channel, Isk-related family, member 3	2.0	$6x10^{-4}$
**KLF4**	Kruppel-like factor 4 (gut)	2.1	$6x10^{-4}$
**MIA3**	melanoma inhibitory activity family, member 3	1.5	$6x10^{-4}$
**PDIA5**	protein disulfide isomerase family A, member 5	1.7	$6x10^{-4}$
HEPACAM2	HEPACAM family member 2	2.5	$6x10^{-4}$
**LRRFIP2**	leucine rich repeat (in FLII) interacting protein 2	1.3	$7x10^{-4}$
SLC31A1	solute carrier family 31 (copper transporters), member 1	1.8	$7x10^{-4}$
FAM171A1	family with sequence similarity 171, member A1	1.7	$7x10^{-4}$
**GALNT6**	UDP-N-acetyl-alpha-D-galactosamine:polypeptide N-acetylgalactosaminyltransferase 6 (GalNAc-T6)	2.4	$8x10^{-4}$
**EIF2AK3**	eukaryotic translation initiation factor 2-alpha kinase 3	1.6	$1x10^{-3}$
**FUT3**	fucosyltransferase 3 (galactoside 3(4)-L-fucosyltransferase, Lewis blood group)	2.1	$1x10^{-3}$
MAGED2	melanoma antigen family D, 2	1.5	$1x10^{-3}$
GMDS	GDP-mannose 4,6-dehydratase	2.3	$2x10^{-3}$
**GALNT7**	UDP-N-acetyl-alpha-D-galactosamine:polypeptide N-acetylgalactosaminyltransferase 7 (GalNAc-T7)	1.8	$2x10^{-3}$
LIMA1	LIM domain and actin binding 1	1.5	$2x10^{-3}$
PP14571	similar to hCG1777210	2.8	$2x10^{-3}$
**DGKA**	diacylglycerol kinase, alpha 80kDa	1.7	$2x10^{-3}$
FOLH1	folate hydrolase (prostate-specific membrane antigen) 1	2.7	$2x10^{-3}$
PRR15	proline rich 15	1.7	$2x10^{-3}$
HDAC1	histone deacetylase 1	1.6	$2x10^{-3}$
**TPD52**	tumor protein D52	1.5	$2x10^{-3}$
**UBE2Q1**	ubiquitin-conjugating enzyme E2Q family member 1	1.6	$2x10^{-3}$

Supplemental Table II. Genes Differently Expressed Between Nonsmoker-high MUC5AC Expressors Compared to Nonsmoker-low MUC5AC Expressors (cont., page 2)

Gene symbol[1]	Gene name	Fold-change[2]	p value[3]
SLC7A1	solute carrier family 7 (cationic amino acid transporter, y+ system), member 1	1.7	$2x10^{-3}$
TRADD	TNFRSF1A-associated via death domain	1.4	$2x10^{-3}$
BAG3	BCL2-associated athanogene 3	1.5	$2x10^{-3}$
BARD1	BRCA1 associated RING domain 1	1.7	$2x10^{-3}$
FAM114A1	family with sequence similarity 114, member A1	1.5	$2x10^{-3}$
JTB	jumping translocation breakpoint	1.4	$2x10^{-3}$
RAP1GAP	RAP1 GTPase activating protein	1.9	$2x10^{-3}$
SLC16A9	solute carrier family 16, member 9 (monocarboxylic acid transporter 9)	2.5	$2x10^{-3}$
S100P	S100 calcium binding protein P	3.0	$3x10^{-3}$
PPFIBP2	PTPRF interacting protein, binding protein 2 (liprin beta 2)	1.5	$3x10^{-3}$
HDAC9	histone deacetylase 9	2.0	$3x10^{-3}$
TP53INP2	tumor protein p53 inducible nuclear protein 2	1.8	$3x10^{-3}$
**GALNT4**	UDP-N-acetyl-alpha-D-galactosamine:polypeptide N-acetylgalactosaminyltransferase 4 (GalNAc-T4)	1.7	$3x10^{-3}$
XPNPEP1	X-prolyl aminopeptidase (aminopeptidase P) 1, soluble	1.4	$3x10^{-3}$
**PRSS23**	protease, serine, 23	1.8	$3x10^{-3}$
**CLCA2**	chloride channel accessory 2	2.6	$4x10^{-3}$
**SYTL2**	synaptotagmin-like 2	1.6	$4x10^{-3}$
FKBP11	FK506 binding protein 11, 19 kDa	2.0	$4x10^{-3}$
**EDEM3**	ER degradation enhancer, mannosidase alpha-like 3	1.6	$4x10^{-3}$
CLDN10	claudin 10	2.0	$5x10^{-3}$
STK39	serine threonine kinase 39 (STE20/SPS1 homolog, yeast)	1.5	$5x10^{-3}$
BCCIP	BRCA2 and CDKN1A interacting protein	1.6	$5x10^{-3}$
**ITPR3**	inositol 1,4,5-triphosphate receptor, type 3	1.6	$5x10^{-3}$
MCM9	minichromosome maintenance complex component 9	1.3	$5x10^{-3}$
PTGFRN	prostaglandin F2 receptor negative regulator	1.4	$5x10^{-3}$
CNKSR3	CNKSR family member 3	1.5	$5x10^{-3}$
ZNF12	zinc finger protein 12	1.5	$5x10^{-3}$
TOB2	transducer of ERBB2, 2	1.6	$6x10^{-3}$
B4GALT4	UDP-Gal:betaGlcNAc beta 1,4- galactosyltransferase, polypeptide 4	1.7	$6x10^{-3}$
ELF3	E74-like factor 3 (ets domain transcription factor, epithelial-specific)	1.5	$6x10^{-3}$
**GALNT12**	UDP-N-acetyl-alpha-D-galactosamine:polypeptide N-acetylgalactosaminyltransferase 12 (GalNAc-T12)	1.5	$6x10^{-3}$
LAYN	layilin	1.6	$6x10^{-3}$
LOXL4	lysyl oxidase-like 4	1.8	$6x10^{-3}$
**MAPK13**	mitogen-activated protein kinase 13	1.5	$6x10^{-3}$
MCF2L	MCF.2 cell line derived transforming sequence-like	1.6	$6x10^{-3}$
**SERPINB4**	serpin peptidase inhibitor, clade B (ovalbumin), member 4	3.3	$6x10^{-3}$
VSIG2	V-set and immunoglobulin domain containing 2	2.1	$6x10^{-3}$
ZNF750	zinc finger protein 750	1.7	$6x10^{-3}$
PTER	phosphotriesterase related	1.6	$6x10^{-3}$
**ST8SIA1**	ST8 alpha-N-acetyl-neuraminide alpha-2,8-sialyltransferase 1	1.9	$6x10^{-3}$

Supplemental Table II. Genes Differently Expressed Between Nonsmoker-high MUC5AC Expressors Compared to Nonsmoker-low MUC5AC Expressors (cont., page 3)

Gene symbol[1]	Gene name	Fold-change[2]	p value[3]
PSMG3	proteasome (prosome, macropain) assembly chaperone 3	1.4	$7x10^{-3}$
NAV1	neuron navigator 1	1.8	$7x10^{-3}$
B4GALT5	UDP-Gal:betaGlcNAc beta 1,4- galactosyltransferase, polypeptide 5	1.6	$7x10^{-3}$
**CKAP4**	cytoskeleton-associated protein 4	1.8	$7x10^{-3}$
**SEC31A**	SEC31 homolog A (S. cerevisiae)	1.3	$7x10^{-3}$
ALG14	asparagine-linked glycosylation 14 homolog (S. cerevisiae)	1.3	$7x10^{-3}$
TMPRSS4	transmembrane protease, serine 4	2.2	$7x10^{-3}$
SLC44A1	solute carrier family 44, member 1	1.4	$7x10^{-3}$
C12orf23	chromosome 12 open reading frame 23	1.5	$7x10^{-3}$
MORC4	MORC family CW-type zinc finger 4	1.5	$8x10^{-3}$
TSPAN8	tetraspanin 8	1.8	$8x10^{-3}$
CANT1	calcium activated nucleotidase 1	1.5	$8x10^{-3}$
CEACAM6	carcinoembryonic antigen-related cell adhesion molecule 6 (non-specific cross reacting antigen)	2.2	$8x10^{-3}$
PARVA	parvin, alpha	1.4	$8x10^{-3}$
FAM3D	family with sequence similarity 3, member D	1.9	$8x10^{-3}$
POM121 /// POM121C	POM121 membrane glycoprotein (rat) /// POM121 membrane glyco-protein C	1.6	$8x10^{-3}$
SERPINB2	serpin peptidase inhibitor, clade B (ovalbumin), member 2	3.5	$8x10^{-3}$
ZNF689	zinc finger protein 689	1.5	$8x10^{-3}$
FBXL7	F-box and leucine-rich repeat protein 7	2.1	$9x10^{-3}$
UGT8	UDP glycosyltransferase 8	2.1	$9x10^{-3}$
AUTS2	autism susceptibility candidate 2	1.5	$9x10^{-3}$
**PAM**	peptidylglycine alpha-amidating monooxygenase	1.8	$9x10^{-3}$
SWAP70	SWAP-70 protein	1.4	$9x10^{-3}$
TRIM26	tripartite motif-containing 26	1.4	$1x10^{-2}$
KBTBD7	kelch repeat and BTB (POZ) domain containing 7	1.3	$1x10^{-2}$
**MPPE1**	Metallophosphoesterase 1	1.7	$1x10^{-2}$
**GSN**	gelsolin (amyloidosis, Finnish type)	2.1	$1x10^{-2}$
LPCAT4	lysophosphatidylcholine acyltransferase 4	1.6	$1x10^{-2}$
FAM110C	family with sequence similarity 110, member C	1.8	$1x10^{-2}$
GNPNAT1	glucosamine-phosphate N-acetyltransferase 1	1.6	$1x10^{-2}$
SECISBP2	SECIS binding protein 2	1.4	$1x10^{-2}$
KRT10	keratin 10	1.4	$1x10^{-2}$
SERPINB5	serpin peptidase inhibitor, clade B (ovalbumin), member 5	2.6	$1x10^{-2}$
TSHZ2	Teashirt zinc finger homeobox 2	1.7	$1x10^{-2}$
ZBTB7C	zinc finger and BTB domain containing 7C	1.5	$1x10^{-2}$
**GABRP**	gamma-aminobutyric acid (GABA) A receptor, pi	2.2	$1x10^{-2}$
RUSC1	RUN and SH3 domain containing 1	1.3	$1x10^{-2}$
GOSR1	golgi SNAP receptor complex member 1	1.3	$1x10^{-2}$
**KDELR2**	KDEL (Lys-Asp-Glu-Leu) endoplasmic reticulum protein retention receptor 2	1.5	$1x10^{-2}$
FAM129A	family with sequence similarity 129, member A	1.8	$1x10^{-2}$
PAK1	p21 protein (Cdc42/Rac)-activated kinase 1	1.2	$1x10^{-2}$
SDC4	syndecan 4	1.5	$1x10^{-2}$

Supplemental Table II. Genes Differently Expressed Between Nonsmoker-high MUC5AC Expressors Compared to Nonsmoker-low MUC5AC Expressors (cont., page 4)

Gene symbol[1]	Gene name	Fold-change[2]	p value[3]
SFN	stratifin	1.8	$1x10^{-2}$
SMARCC1	SWI/SNF related, matrix associated, actin dependent regulator of chromatin, subfamily c, member 1	1.4	$1x10^{-2}$
THRB	thyroid hormone receptor, beta (erythroblastic leukemia viral (v-erb-a) oncogene homolog 2, avian)	1.6	$1x10^{-2}$
DNAJC22	DnaJ (Hsp40) homolog, subfamily C, member 22	1.5	$1x10^{-2}$
S100A16	S100 calcium binding protein A16	1.8	$1x10^{-2}$
**SOX2**	SRY (sex determining region Y)-box 2	1.4	$1x10^{-2}$
**CTSC**	cathepsin C	1.7	$1x10^{-2}$
RDH10	retinol dehydrogenase 10 (all-trans)	1.6	$1x10^{-2}$
SLC26A2	solute carrier family 26 (sulfate transporter), member 2	1.7	$1x10^{-2}$
ZNF827	Zinc finger protein 827	1.3	$1x10^{-2}$
CXCL1	chemokine (C-X-C motif) ligand 1 (melanoma growth stimulating activity, alpha)	1.9	$1x10^{-2}$
ABHD2	abhydrolase domain containing 2	1.6	$1x10^{-2}$
GOLSYN	Golgi-localized protein	1.5	$1x10^{-2}$
MYLIP	myosin regulatory light chain interacting protein	1.4	$1x10^{-2}$
OSTalpha	organic solute transporter alpha	1.8	$1x10^{-2}$
TIMM13	translocase of inner mitochondrial membrane 13 homolog (yeast)	1.5	$1x10^{-2}$
WFDC2	WAP four-disulfide core domain 2	1.7	$1x10^{-2}$
CKMT1A /// CKMT1B	creatine kinase, mitochondrial 1A /// creatine kinase, mitochondrial 1B	1.6	$1x10^{-2}$
ADAMTSL3	ADAMTS-like 3	1.5	$1x10^{-2}$
IER3	immediate early response 3	2.7	$1x10^{-2}$
PDLIM5	PDZ and LIM domain 5	1.4	$1x10^{-2}$
AKR1A1	aldo-keto reductase family 1, member A1 (aldehyde reductase)	1.4	$1x10^{-2}$
BLMH	bleomycin hydrolase	1.4	$1x10^{-2}$
MPHOSPH10	M-phase phosphoprotein 10 (U3 small nucleolar ribonucleoprotein)	1.3	$1x10^{-2}$
USP22	ubiquitin specific peptidase 22	1.4	$1x10^{-2}$
ZDHHC16	zinc finger, DHHC-type containing 16	1.3	$1x10^{-2}$
**CASK**	calcium/calmodulin-dependent serine protein kinase (MAGUK family)	1.3	$1x10^{-2}$
DHRS9	dehydrogenase/reductase (SDR family) member 9	1.8	$1x10^{-2}$
PPAPDC2	phosphatidic acid phosphatase type 2 domain containing 2	1.5	$1x10^{-2}$
SLC9A3R1	solute carrier family 9 (sodium/hydrogen exchanger), member 3 regulator 1	1.6	$1x10^{-2}$
**ARF4**	ADP-ribosylation factor 4	1.3	$1x10^{-2}$
BACE2	beta-site APP-cleaving enzyme 2	1.8	$2x10^{-2}$
BRD2	bromodomain containing 2	1.5	$2x10^{-2}$
CTBP1	C-terminal binding protein 1	1.4	$2x10^{-2}$
CTTNBP2NL	CTTNBP2 N-terminal like	1.7	$2x10^{-2}$
FXC1	fracture callus 1 homolog (rat)	1.4	$2x10^{-2}$
OAT	ornithine aminotransferase (gyrate atrophy)	1.7	$2x10^{-2}$
OBFC1	oligonucleotide/oligosaccharide-binding fold containing 1	1.5	$2x10^{-2}$
PRSS8	protease, serine, 8	1.8	$2x10^{-2}$

Supplemental Table II. Genes Differently Expressed Between Nonsmoker-high MUC5AC Expressors Compared to Nonsmoker-low MUC5AC Expressors (cont., page 5)

Gene symbol[1]	Gene name	Fold-change[2]	p value[3]
**SYTL4**	synaptotagmin-like 4	1.7	$2x10^{-2}$
WWP1	WW domain containing E3 ubiquitin protein ligase 1	1.4	$2x10^{-2}$
SGPL1	sphingosine-1-phosphate lyase 1	1.3	$2x10^{-2}$
**SYTL5**	synaptotagmin-like 5	1.6	$2x10^{-2}$
F3	coagulation factor III (thromboplastin, tissue factor)	1.9	$2x10^{-2}$
**RAB27B**	RAB27B, member RAS oncogene family	1.6	$2x10^{-2}$
FAM108C1	family with sequence similarity 108, member C1	1.5	$2x10^{-2}$
ACBD3	acyl-Coenzyme A binding domain containing 3	1.3	$2x10^{-2}$
C3orf58	chromosome 3 open reading frame 58	1.3	$2x10^{-2}$
CLMN	calmin (calponin-like, transmembrane)	1.4	$2x10^{-2}$
DHX32	DEAH (Asp-Glu-Ala-His) box polypeptide 32	1.5	$2x10^{-2}$
KLF3	Kruppel-like factor 3 (basic)	1.5	$2x10^{-2}$
KLHL6	kelch-like 6 (Drosophila)	1.9	$2x10^{-2}$
PGM2L1	phosphoglucomutase 2-like 1	1.5	$2x10^{-2}$
RAB11FIP1	RAB11 family interacting protein 1 (class I)	1.5	$2x10^{-2}$
TPSAB1	tryptase alpha/beta 1	5.8	$2x10^{-2}$
TPSB2	tryptase beta 2	5.0	$2x10^{-2}$
TUG1	taurine upregulated 1 (non-protein coding)	1.4	$2x10^{-2}$
UPK1B	uroplakin 1B	3.1	$2x10^{-2}$
ZC3H18	zinc finger CCCH-type containing 18	1.5	$2x10^{-2}$
TBC1D1	TBC1 (tre-2/USP6, BUB2, cdc16) domain family, member 1	1.4	$2x10^{-2}$
**SPDEF**	SAM pointed domain containing ets transcription factor	2.1	$2x10^{-2}$
ATF3	activating transcription factor 3	2.0	$2x10^{-2}$
FAM109B	family with sequence similarity 109, member B	1.8	$2x10^{-2}$
MFSD4	major facilitator superfamily domain containing 4	1.8	$2x10^{-2}$
SDR16C5	short chain dehydrogenase/reductase family 16C, member 5	1.5	$2x10^{-2}$
**SLC12A2**	solute carrier family 12 (sodium/potassium/chloride transporters), member 2	1.6	$2x10^{-2}$
PRR4	proline rich 4 (lacrimal)	3.8	$2x10^{-2}$
BAMBI	BMP and activin membrane-bound inhibitor homolog (Xenopus lae-vis)	1.5	$2x10^{-2}$
C9orf5	chromosome 9 open reading frame 5	1.3	$2x10^{-2}$
NIPAL3	NIPA-like domain containing 3	1.5	$2x10^{-2}$
SERPINB13	serpin peptidase inhibitor, clade B (ovalbumin), member 13	2.0	$2x10^{-2}$
ZNF565	zinc finger protein 565	1.3	$2x10^{-2}$
WIPF2	WAS/WASL interacting protein family, member 2	1.4	$2x10^{-2}$
EGR1	early growth response 1	2.6	$2x10^{-2}$
STK24	serine/threonine kinase 24 (STE20 homolog, yeast)	1.3	$2x10^{-2}$
ASAP2	ArfGAP with SH3 domain, ankyrin repeat and PH domain 2	1.4	$2x10^{-2}$
CXCL17	chemokine (C-X-C motif) ligand 17	1.7	$2x10^{-2}$
DNAJC3	DnaJ (Hsp40) homolog, subfamily C, member 3	1.3	$2x10^{-2}$
EYA2	eyes absent homolog 2 (Drosophila)	1.8	$2x10^{-2}$
FZD5	frizzled homolog 5 (Drosophila)	1.5	$2x10^{-2}$
GSPT1	G1 to S phase transition 1	1.4	$2x10^{-2}$
**ITSN1**	intersectin 1 (SH3 domain protein)	1.5	$2x10^{-2}$

Supplemental Table II. Genes Differently Expressed Between Nonsmoker-high MUC5AC Expressors Compared to Nonsmoker-low MUC5AC Expressors (cont., page 6)

Gene symbol[1]	Gene name	Fold-change[2]	p value[3]
LOC400573	hypothetical gene supported by BC015790; BC041634	1.8	$2x10^{-2}$
NAT10	N-acetyltransferase 10 (GCN5-related)	1.3	$2x10^{-2}$
VTCN1	V-set domain containing T cell activation inhibitor 1	1.7	$2x10^{-2}$
EPS8L1	EPS8-like 1	1.5	$2x10^{-2}$
MTSS1	metastasis suppressor 1	1.4	$2x10^{-2}$
NDE1	nudE nuclear distribution gene E homolog 1 (A. nidulans)	1.5	$2x10^{-2}$
TBC1D8B	TBC1 domain family, member 8B (with GRAM domain)	1.4	$2x10^{-2}$
C9orf152	chromosome 9 open reading frame 152	1.4	$2x10^{-2}$
HM13	histocompatibility (minor) 13	1.5	$2x10^{-2}$
CAPN9	calpain 9	1.6	$2x10^{-2}$
KCTD14	potassium channel tetramerisation domain containing 14	1.7	$2x10^{-2}$
**FUT6**	fucosyltransferase 6 (alpha (1,3) fucosyltransferase)	1.6	$2x10^{-2}$
ALPL	alkaline phosphatase, liver/bone/kidney	2.0	$2x10^{-2}$
**RAB3D**	RAB3D, member RAS oncogene family	1.6	$2x10^{-2}$
BUD13	BUD13 homolog (S. cerevisiae)	1.4	$2x10^{-2}$
TJP1	tight junction protein 1 (zona occludens 1)	1.3	$2x10^{-2}$
TPBG	trophoblast glycoprotein	1.5	$2x10^{-2}$
TTLL12	tubulin tyrosine ligase-like family, member 12	1.5	$2x10^{-2}$
NHSL1	NHS-like 1	1.4	$2x10^{-2}$
HERPUD2	HERPUD family member 2	1.4	$2x10^{-2}$
MAGED1	melanoma antigen family D, 1	1.5	$2x10^{-2}$
RUNX1	runt-related transcription factor 1	1.4	$2x10^{-2}$
NF2	neurofibromin 2 (merlin)	1.4	$2x10^{-2}$
**VPS13D**	vacuolar protein sorting 13 homolog D (S. cerevisiae)	1.5	$2x10^{-2}$
DLG3	discs, large homolog 3 (Drosophila)	1.4	$2x10^{-2}$
PTPRZ1	protein tyrosine phosphatase, receptor-type, Z polypeptide 1	1.7	$2x10^{-2}$
ATP2C2	ATPase, Ca++ transporting, type 2C, member 2	1.7	$2x10^{-2}$
ECE1	endothelin converting enzyme 1	1.4	$2x10^{-2}$
FAF1	Fas (TNFRSF6) associated factor 1	1.4	$2x10^{-2}$
**TSTA3**	tissue specific transplantation antigen P35B	1.4	$2x10^{-2}$
CCDC88C	coiled-coil domain containing 88C	1.4	$2x10^{-2}$
SLC37A3	solute carrier family 37 (glycerol-3-phosphate transporter), member 3	1.4	$2x10^{-2}$
SLC39A11	solute carrier family 39 (metal ion transporter), member 11	1.5	$2x10^{-2}$
TNFAIP3	tumor necrosis factor, alpha-induced protein 3	1.5	$2x10^{-2}$
CHMP7	CHMP family, member 7	1.4	$2x10^{-2}$
GNA11	Guanine nucleotide binding protein (G protein), alpha 11 (Gq class)	1.4	$2x10^{-2}$
GRLF1	glucocorticoid receptor DNA binding factor 1	1.4	$2x10^{-2}$
TSPAN13	tetraspanin 13	1.7	$2x10^{-2}$
TTPAL	tocopherol (alpha) transfer protein-like	1.5	$2x10^{-2}$
INPP1	inositol polyphosphate-1-phosphatase	1.3	$2x10^{-2}$
PHC3	polyhomeotic homolog 3 (Drosophila)	1.3	$2x10^{-2}$
GRTP1	growth hormone regulated TBC protein 1	1.4	$2x10^{-2}$
HNRNPD	heterogeneous nuclear ribonucleoprotein D (AU-rich element RNA binding protein 1, 37kDa)	1.2	$2x10^{-2}$

Supplemental Table II. Genes Differently Expressed Between Nonsmoker-high MUC5AC Expressors Compared to Nonsmoker-low MUC5AC Expressors (cont., page 7)

Gene symbol[1]	Gene name	Fold-change[2]	p value[3]
**PRKCD**	protein kinase C, delta	1.5	$2x10^{-2}$
UROS	uroporphyrinogen III synthase	1.5	$2x10^{-2}$
EEF1D	eukaryotic translation elongation factor 1 delta (guanine nucleotide exchange protein)	1.5	$2x10^{-2}$
PRKAB1	protein kinase, AMP-activated, beta 1 non-catalytic subunit	1.3	$2x10^{-2}$
ALDH18A1	aldehyde dehydrogenase 18 family, member A1	1.4	$2x10^{-2}$
ALDH6A1	aldehyde dehydrogenase 6 family, member A1	1.3	$2x10^{-2}$
**PCLO**	piccolo (presynaptic cytomatrix protein)	1.4	$2x10^{-2}$
RARS	arginyl-tRNA synthetase	2.3	$2x10^{-2}$
YRDC	yrdC domain containing (E. coli)	1.4	$2x10^{-2}$
WHAMM	WAS protein homolog associated with actin, golgi membranes and microtubules	1.4	$3x10^{-2}$
C2CD2	C2 calcium-dependent domain containing 2	1.4	$3x10^{-2}$
ELL2	elongation factor, RNA polymerase II, 2	1.3	$3x10^{-2}$
PIK3R3	phosphoinositide-3-kinase, regulatory subunit 3 (gamma)	1.4	$3x10^{-2}$
SPG11	spastic paraplegia 11 (autosomal recessive)	1.3	$3x10^{-2}$
TES	testis derived transcript (3 LIM domains)	1.4	$3x10^{-2}$
TM9SF3	transmembrane 9 superfamily member 3	1.3	$3x10^{-2}$
ENTPD3	ectonucleoside triphosphate diphosphohydrolase 3	1.5	$3x10^{-2}$
CYP2C9	cytochrome P450, family 2, subfamily C, polypeptide 9	1.7	$3x10^{-2}$
MAFK	v-maf musculoaponeurotic fibrosarcoma oncogene homolog K (avian)	1.3	$3x10^{-2}$
AACS	acetoacetyl-CoA synthetase	1.3	$3x10^{-2}$
B3GNT9	UDP-GlcNAc:betaGal beta-1,3-N-acetylglucosaminyltransferase 9	1.4	$3x10^{-2}$
CAPN5	calpain 5	1.6	$3x10^{-2}$
**CHST6**	carbohydrate (N-acetylglucosamine 6-O) sulfotransferase 6	1.6	$3x10^{-2}$
ELL3	elongation factor RNA polymerase II-like 3	1.3	$3x10^{-2}$
HDLBP	high density lipoprotein binding protein	1.6	$3x10^{-2}$
IRGQ	immunity-related GTPase family, Q	1.3	$3x10^{-2}$
JUN	jun oncogene	1.7	$3x10^{-2}$
JUNB	jun B proto-oncogene	2.4	$3x10^{-2}$
LYRM2	LYR motif containing 2	1.3	$3x10^{-2}$
ODZ4	odz, odd Oz/ten-m homolog 4 (Drosophila)	1.5	$3x10^{-2}$
**PLA2G4A**	phospholipase A2, group IVA (cytosolic, calcium-dependent)	1.7	$3x10^{-2}$
SIK1	salt-inducible kinase 1	2.1	$3x10^{-2}$
SNX19	sorting nexin 19	1.3	$3x10^{-2}$
SNX5	sorting nexin 5	1.4	$3x10^{-2}$
TRAK1	trafficking protein, kinesin binding 1	1.6	$3x10^{-2}$
VPS37B	vacuolar protein sorting 37 homolog B (S. cerevisiae)	1.5	$3x10^{-2}$
PARP11	poly (ADP-ribose) polymerase family, member 11	1.4	$3x10^{-2}$
SRPK1	SFRS protein kinase 1	1.3	$3x10^{-2}$
CREB3L4	cAMP responsive element binding protein 3-like 4	1.4	$3x10^{-2}$
EZR	ezrin	1.2	$3x10^{-2}$

Supplemental Table II. Genes Differently Expressed Between Nonsmoker-high MUC5AC Expressors Compared to Nonsmoker-low MUC5AC Expressors (cont., page 8)

Gene symbol[1]	Gene name	Fold-change[2]	p value[3]
FAM21A /// FAM21B /// FAM21C /// FAM21D	family with sequence similarity 21, member A /// family with sequence similarity 21, member B /// family with sequence similarity 21, member C /// family with sequence similarity 21, member D	1.4	$3x10^{-2}$
GADD45G	growth arrest and DNA-damage-inducible, gamma	1.5	$3x10^{-2}$
PDE8B	phosphodiesterase 8B	1.6	$3x10^{-2}$
UBR2	ubiquitin protein ligase E3 component n-recognin 2	1.3	$3x10^{-2}$
EIF4G2	eukaryotic translation initiation factor 4 gamma, 2	1.2	$3x10^{-2}$
ALDH1A3	aldehyde dehydrogenase 1 family, member A3	1.7	$3x10^{-2}$
DMRT2	doublesex and mab-3 related transcription factor 2	1.6	$3x10^{-2}$
SFRS4	splicing factor, arginine/serine-rich 4	1.3	$3x10^{-2}$
ENOX2	ecto-NOX disulfide-thiol exchanger 2	1.3	$3x10^{-2}$
FUNDC1	FUN14 domain containing 1	1.5	$3x10^{-2}$
SLC37A1	solute carrier family 37 (glycerol-3-phosphate transporter), member 1	1.5	$3x10^{-2}$
NCK2	NCK adaptor protein 2	1.4	$3x10^{-2}$
KPNA4	karyopherin alpha 4 (importin alpha 3)	1.3	$3x10^{-2}$
**SEC22B**	SEC22 vesicle trafficking protein homolog B (S. cerevisiae)	1.3	$3x10^{-2}$
ZNF295	zinc finger protein 295	1.4	$3x10^{-2}$
GART	phosphoribosylglycinamide formyltransferase, phosphoribosylglycinamide synthetase, phosphoribosylaminoimidazole synthetase	1.3	$3x10^{-2}$
**KRAS**	v-Ki-ras2 Kirsten rat sarcoma viral oncogene homolog	1.3	$3x10^{-2}$
ESRP1	epithelial splicing regulatory protein 1	1.3	$3x10^{-2}$
ZNRF1	zinc and ring finger 1	1.4	$3x10^{-2}$
**RIMS1**	regulating synaptic membrane exocytosis 1	1.6	$3x10^{-2}$
ST14	suppression of tumorigenicity 14 (colon carcinoma)	1.9	$3x10^{-2}$
KIF16B	kinesin family member 16B	1.5	$3x10^{-2}$
**ATP6V0A4**	ATPase, H+ transporting, lysosomal V0 subunit a4	1.6	$3x10^{-2}$
CTBP2	C-terminal binding protein 2	1.3	$3x10^{-2}$
DPYSL3	dihydropyrimidinase-like 3	2.1	$3x10^{-2}$
PHF20L1	PHD finger protein 20-like 1	1.3	$3x10^{-2}$
PRPSAP1	phosphoribosyl pyrophosphate synthetase-associated protein 1	1.3	$3x10^{-2}$
RAB6A /// RAB6C	RAB6A, member RAS oncogene family /// RAB6C, member RAS oncogene family	1.3	$3x10^{-2}$
RBM15B	RNA binding motif protein 15B	1.4	$3x10^{-2}$
RIBC1	RIB43A domain with coiled-coils 1	1.6	$3x10^{-2}$
**SCNN1A**	sodium channel, nonvoltage-gated 1 alpha	1.4	$3x10^{-2}$
SUOX	sulfite oxidase	1.4	$3x10^{-2}$
ZNF805	zinc finger protein 805	1.4	$3x10^{-2}$
SEC16A	SEC16 homolog A (S. cerevisiae)	1.4	$3x10^{-2}$
OLFML2A	olfactomedin-like 2A	1.9	$3x10^{-2}$
SLK	STE20-like kinase (yeast)	1.4	$3x10^{-2}$
CADPS2	Ca++-dependent secretion activator 2	1.4	$3x10^{-2}$
KIAA0746	KIAA0746 protein	1.6	$3x10^{-2}$
KIT	v-kit Hardy-Zuckerman 4 feline sarcoma viral oncogene homolog	2.5	$3x10^{-2}$

Supplemental Table II. Genes Differently Expressed Between Nonsmoker-high MUC5AC Expressors Compared to Nonsmoker-low MUC5AC Expressors (cont., page 9)

Gene symbol[1]	Gene name	Fold-change[2]	p value[3]
METTL13	methyltransferase like 13	1.3	$3x10^{-2}$
PDXDC1	pyridoxal-dependent decarboxylase domain containing 1	1.5	$3x10^{-2}$
TUBB2A	tubulin, beta 2A	1.5	$3x10^{-2}$
ZNF143	zinc finger protein 143	1.3	$3x10^{-2}$
ADORA2B	adenosine A2b receptor	1.5	$3x10^{-2}$
C20orf72	chromosome 20 open reading frame 72	1.4	$3x10^{-2}$
hCG_1817306	hypothetical LOC100130691	1.4	$3x10^{-2}$
IRF6	interferon regulatory factor 6	1.5	$3x10^{-2}$
MRAP2	melanocortin 2 receptor accessory protein 2	1.6	$3x10^{-2}$
NGRN	neugrin, neurite outgrowth associated	1.4	$3x10^{-2}$
**ST6GAL1**	ST6 beta-galactosamide alpha-2,6-sialyltranferase 1	1.6	$3x10^{-2}$
OSBPL2	oxysterol binding protein-like 2	1.4	$3x10^{-2}$
POLR2C	polymerase (RNA) II (DNA directed) polypeptide C, 33kDa	1.3	$3x10^{-2}$
SLC6A14	solute carrier family 6 (amino acid transporter), member 14	1.9	$3x10^{-2}$
B3GNT3	UDP-GlcNAc:betaGal beta-1,3-N-acetylglucosaminyltransferase 3	1.6	$3x10^{-2}$
MFSD6	major facilitator superfamily domain containing 6	1.3	$3x10^{-2}$
**MYO5C**	myosin VC	1.3	$3x10^{-2}$
LAPTM4B	lysosomal protein transmembrane 4 beta	1.6	$3x10^{-2}$
UST	uronyl-2-sulfotransferase	1.4	$3x10^{-2}$
S100A14	S100 calcium binding protein A14	1.8	$3x10^{-2}$
C10orf81	chromosome 10 open reading frame 81	1.7	$3x10^{-2}$
DEFB1	defensin, beta 1	2.0	$3x10^{-2}$
FERMT1	fermitin family homolog 1 (Drosophila)	1.6	$3x10^{-2}$
GSR	glutathione reductase	1.5	$3x10^{-2}$
LASP1	LIM and SH3 protein 1	1.3	$3x10^{-2}$
ME3	malic enzyme 3, NADP(+)-dependent, mitochondrial	1.4	$3x10^{-2}$
MKNK2	MAP kinase interacting serine/threonine kinase 2	1.6	$3x10^{-2}$
**PLCE1**	phospholipase C, epsilon 1	1.5	$3x10^{-2}$
RREB1	ras responsive element binding protein 1	1.3	$3x10^{-2}$
STIM2	stromal interaction molecule 2	1.4	$3x10^{-2}$
ZNF212	zinc finger protein 212	1.3	$3x10^{-2}$
CCBL1	cysteine conjugate-beta lyase, cytoplasmic	1.4	$3x10^{-2}$
UBE2Q2	ubiquitin-conjugating enzyme E2Q family member 2	1.3	$3x10^{-2}$
UPF1	UPF1 regulator of nonsense transcripts homolog (yeast)	1.4	$3x10^{-2}$
**KIF5B**	kinesin family member 5B	1.3	$3x10^{-2}$
SIPA1L1	signal-induced proliferation-associated 1 like 1	1.5	$3x10^{-2}$
GPR56	G protein-coupled receptor 56	1.6	$3x10^{-2}$
ILDR1	immunoglobulin-like domain containing receptor 1	1.4	$3x10^{-2}$
KIAA1324	KIAA1324	1.6	$3x10^{-2}$
SLC45A3	solute carrier family 45, member 3	1.7	$3x10^{-2}$
FGF13	fibroblast growth factor 13	2.0	$3x10^{-2}$
MDM2	Mdm2 p53 binding protein homolog (mouse)	1.4	$3x10^{-2}$
FAM118B	family with sequence similarity 118, member B	1.4	$4x10^{-2}$
ORMDL3	ORM1-like 3 (S. cerevisiae)	1.4	$4x10^{-2}$

Supplemental Table II. Genes Differently Expressed Between Nonsmoker-high MUC5AC Expressors Compared to Nonsmoker-low MUC5AC Expressors (cont., page 10)

Gene symbol[1]	Gene name	Fold-change[2]	p value[3]
RHBDL2	rhomboid, veinlet-like 2 (Drosophila)	1.6	$4x10^{-2}$
BEX5	brain expressed, X-linked 5	1.7	$4x10^{-2}$
UBE2A	ubiquitin-conjugating enzyme E2A (RAD6 homolog)	1.3	$4x10^{-2}$
GNAL	guanine nucleotide binding protein (G protein), alpha activating activity polypeptide, olfactory type	2.1	$4x10^{-2}$
KIAA1211	KIAA1211	2.7	$4x10^{-2}$
NTS	neurotensin	4.1	$4x10^{-2}$
RSL1D1	ribosomal L1 domain containing 1	1.3	$4x10^{-2}$
MYO18A /// TIAF1	myosin XVIIIA /// TGFB1-induced anti-apoptotic factor 1	1.5	$4x10^{-2}$
TSR1	TSR1, 20S rRNA accumulation, homolog (S. cerevisiae)	1.5	$4x10^{-2}$
ZDHHC9	zinc finger, DHHC-type containing 9	1.5	$4x10^{-2}$
DEDD	death effector domain containing	1.2	$4x10^{-2}$
ERMP1	endoplasmic reticulum metallopeptidase 1	1.4	$4x10^{-2}$
NET1	neuroepithelial cell transforming 1	1.5	$4x10^{-2}$
ATP7B	ATPase, Cu++ transporting, beta polypeptide	1.6	$4x10^{-2}$
CSRNP2	cysteine-serine-rich nuclear protein 2	1.3	$4x10^{-2}$
**HES1**	hairy and enhancer of split 1, (Drosophila)	1.7	$4x10^{-2}$
IL17RD	interleukin 17 receptor D	1.5	$4x10^{-2}$
MON2	MON2 homolog (S. cerevisiae)	1.2	$4x10^{-2}$
PAICS	phosphoribosylaminoimidazole carboxylase, phosphoribosylaminoimidazole succinocarboxamide synthetase	1.3	$4x10^{-2}$
PLLP	plasma membrane proteolipid (plasmolipin)	1.5	$4x10^{-2}$
USP48	ubiquitin specific peptidase 48	1.3	$4x10^{-2}$
**MYO5B**	myosin VB	1.4	$4x10^{-2}$
FOXN3	forkhead box N3	1.5	$4x10^{-2}$
KIAA0415	KIAA0415	1.4	$4x10^{-2}$
PWP1	PWP1 homolog (S. cerevisiae)	1.2	$4x10^{-2}$
**SCIN**	scinderin	1.5	$4x10^{-2}$
CHMP2B	chromatin modifying protein 2B	1.3	$4x10^{-2}$
LOC644172 /// MAPK8IP1	mitogen-activated protein kinase 8 interacting protein 1 pseudogene /// mitogen-activated protein kinase 8 interacting protein 1	1.5	$4x10^{-2}$
BCORL1	BCL6 co-repressor-like 1	1.4	$4x10^{-2}$
CDH26	cadherin-like 26	1.7	$4x10^{-2}$
LEPROT	leptin receptor overlapping transcript	1.5	$4x10^{-2}$
NR4A2	nuclear receptor subfamily 4, group A, member 2	1.9	$4x10^{-2}$
UTP14A	UTP14, U3 small nucleolar ribonucleoprotein, homolog A (yeast)	1.3	$4x10^{-2}$
AGPAT3	1-acylglycerol-3-phosphate O-acyltransferase 3	1.3	$4x10^{-2}$
AKAP1	A kinase (PRKA) anchor protein 1	1.5	$4x10^{-2}$
FLII	flightless I homolog (Drosophila)	1.4	$4x10^{-2}$
HUNK	hormonally up-regulated Neu-associated kinase	1.6	$4x10^{-2}$
LPAR1	lysophosphatidic acid receptor 1	1.5	$4x10^{-2}$
PPP2R3A	protein phosphatase 2 (formerly 2A), regulatory subunit B", alpha	1.4	$4x10^{-2}$
**RPS6KA3**	ribosomal protein S6 kinase, 90kDa, polypeptide 3	1.4	$4x10^{-2}$
STYK1	serine/threonine/tyrosine kinase 1	1.5	$4x10^{-2}$
VPS35	vacuolar protein sorting 35 homolog (S. cerevisiae)	1.4	$4x10^{-2}$

Supplemental Table II. Genes Differently Expressed Between Nonsmoker-high MUC5AC Expressors Compared to Nonsmoker-low MUC5AC Expressors (cont., page 11)

Gene symbol[1]	Gene name	Fold-change[2]	p value[3]
SVIP	small VCP/p97-interacting protein	1.4	$4x10^{-2}$
LOC389023	hypothetical gene supported by BC032913; BC048425	1.7	$4x10^{-2}$
ADRA2A	adrenergic, alpha-2A-, receptor	2.0	$4x10^{-2}$
KCTD1	potassium channel tetramerisation domain containing 1	1.4	$4x10^{-2}$
MEIS3P1	Meis homeobox 3 pseudogene 1	1.4	$4x10^{-2}$
METTL4	methyltransferase like 4	1.2	$4x10^{-2}$
NIPSNAP1	nipsnap homolog 1 (C. elegans)	1.5	$4x10^{-2}$
RUNX2	runt-related transcription factor 2	1.5	$4x10^{-2}$
TLE1	transducin-like enhancer of split 1 (E(sp1) homolog, Drosophila)	1.5	$4x10^{-2}$
ZDHHC3	zinc finger, DHHC-type containing 3	1.3	$4x10^{-2}$
DMRT3	doublesex and mab-3 related transcription factor 3	1.6	$4x10^{-2}$
MRPS22	mitochondrial ribosomal protein S22	1.3	$4x10^{-2}$
GPRC5C	G protein-coupled receptor, family C, group 5, member C	1.4	$4x10^{-2}$
LOC339290	hypothetical LOC339290	1.4	$4x10^{-2}$
NOP14	NOP14 nucleolar protein homolog (yeast)	1.3	$4x10^{-2}$
SLC2A10	solute carrier family 2 (facilitated glucose transporter), member 10	1.6	$4x10^{-2}$
VEZF1	vascular endothelial zinc finger 1	1.3	$4x10^{-2}$
EPN3	epsin 3	1.6	$4x10^{-2}$
OCIAD1	OCIA domain containing 1	1.3	$4x10^{-2}$
FLJ23867 /// QSOX1	hypothetical protein FLJ23867 /// quiescin Q6 sulfhydryl oxidase 1	1.4	$4x10^{-2}$
PABPC3	poly(A) binding protein, cytoplasmic 3	1.3	$4x10^{-2}$
PRKDC	protein kinase, DNA-activated, catalytic polypeptide	1.4	$4x10^{-2}$
SLC1A5	solute carrier family 1 (neutral amino acid transporter), member 5	1.6	$4x10^{-2}$
SRPRB	signal recognition particle receptor, B subunit	1.4	$4x10^{-2}$
SYNJ2	synaptojanin 2	1.3	$4x10^{-2}$
DIAPH2	diaphanous homolog 2 (Drosophila)	1.6	$4x10^{-2}$
FAM59A	family with sequence similarity 59, member A	1.4	$4x10^{-2}$
**SURF4**	surfeit 4	1.6	$4x10^{-2}$
VPS37C	vacuolar protein sorting 37 homolog C (S. cerevisiae)	1.3	$4x10^{-2}$
ARRDC2	arrestin domain containing 2	1.3	$4x10^{-2}$
TJP3	tight junction protein 3 (zona occludens 3)	1.8	$4x10^{-2}$
BSPRY	B-box and SPRY domain containing	1.4	$4x10^{-2}$
TSPAN3	tetraspanin 3	1.3	$4x10^{-2}$
BRPF3	bromodomain and PHD finger containing, 3	1.3	$4x10^{-2}$
C16orf88	chromosome 16 open reading frame 88	1.3	$4x10^{-2}$
**ERGIC1**	endoplasmic reticulum-golgi intermediate compartment (ERGIC) 1	1.7	$4x10^{-2}$
INTS4	integrator complex subunit 4	1.2	$4x10^{-2}$
SAR1B	SAR1 homolog B (S. cerevisiae)	1.3	$4x10^{-2}$
TMEM87B	transmembrane protein 87B	1.3	$4x10^{-2}$
CNOT2	CCR4-NOT transcription complex, subunit 2	1.2	$4x10^{-2}$
SERINC2	serine incorporator 2	1.8	$4x10^{-2}$
CLPTM1L	CLPTM1-like	1.4	$4x10^{-2}$
TCP1	t-complex 1	1.4	$4x10^{-2}$
HISPPD2A	histidine acid phosphatase domain containing 2A	1.4	$4x10^{-2}$

Supplemental Table II. Genes Differently Expressed Between Nonsmoker-high MUC5AC Expressors Compared to Nonsmoker-low MUC5AC Expressors (cont., page 12)

Gene symbol[1]	Gene name	Fold-change[2]	p value[3]
INPP4B	inositol polyphosphate-4-phosphatase, type II, 105kDa	1.7	$4x10^{-2}$
MLEC	malectin	1.4	$4x10^{-2}$
PRMT2	protein arginine methyltransferase 2	1.3	$4x10^{-2}$
UGT2A1 /// UGT2A2	UDP glucuronosyltransferase 2 family, polypeptide A1 /// UDP glucuronosyltransferase 2 family, polypeptide A2	2.6	$4x10^{-2}$
TRAF4	TNF receptor-associated factor 4	1.4	$4x10^{-2}$
TUBG1	tubulin, gamma 1	1.5	$4x10^{-2}$
HK2	hexokinase 2	1.6	$4x10^{-2}$
MBD6	methyl-CpG binding domain protein 6	1.4	$4x10^{-2}$
ZNF318	zinc finger protein 318	1.3	$4x10^{-2}$
BTG2	BTG family, member 2	1.8	$4x10^{-2}$
NUAK1	NUAK family, SNF1-like kinase, 1	1.7	$4x10^{-2}$
RAB38	RAB38, member RAS oncogene family	1.8	$4x10^{-2}$
**SYNJ2BP**	synaptojanin 2 binding protein	1.6	$4x10^{-2}$
UBP1	upstream binding protein 1 (LBP-1a)	1.3	$4x10^{-2}$
ZNF264	zinc finger protein 264	1.3	$4x10^{-2}$
B3GNT2	UDP-GlcNAc:betaGal beta-1,3-N-acetylglucosaminyltransferase 2	1.4	$5x10^{-2}$
CPA3	carboxypeptidase A3 (mast cell)	4.4	$5x10^{-2}$
LOC387723	similar to hCG1648656	1.3	$5x10^{-2}$
SIX2	SIX homeobox 2	1.7	$5x10^{-2}$
ADAMTS17	ADAM metallopeptidase with thrombospondin type 1 motif, 17	1.8	$5x10^{-2}$
FARP1	FERM, RhoGEF (ARHGEF) and pleckstrin domain protein 1 (chondrocyte-derived)	1.4	$5x10^{-2}$
NAGLU	N-acetylglucosaminidase, alpha-	1.4	$5x10^{-2}$
AKIRIN1	akirin 1	1.4	$5x10^{-2}$
C4orf33	chromosome 4 open reading frame 33	1.9	$5x10^{-2}$
BAG1	BCL2-associated athanogene	1.5	$5x10^{-2}$
BBX	bobby sox homolog (Drosophila)	1.3	$5x10^{-2}$
CAPN1	calpain 1, (mu/I) large subunit	1.5	$5x10^{-2}$
OSBPL10	oxysterol binding protein-like 10	1.4	$5x10^{-2}$
UGCG	UDP-glucose ceramide glucosyltransferase	1.3	$5x10^{-2}$
SMS	spermine synthase	1.5	$5x10^{-2}$
GNL2	guanine nucleotide binding protein-like 2 (nucleolar)	1.4	$5x10^{-2}$
KCTD2	potassium channel tetramerisation domain containing 2	1.3	$5x10^{-2}$
LONP2	lon peptidase 2, peroxisomal	1.4	$5x10^{-2}$
MPZL3	myelin protein zero-like 3	1.4	$5x10^{-2}$
SLC4A8	solute carrier family 4, sodium bicarbonate cotransporter, member 8	1.5	$5x10^{-2}$
ZKSCAN5	zinc finger with KRAB and SCAN domains 5	1.3	$5x10^{-2}$
PPM2C	protein phosphatase 2C, magnesium-dependent, catalytic subunit	1.5	$5x10^{-2}$
FAM58A	family with sequence similarity 58, member A	1.2	$5x10^{-2}$
FBXL18	F-box and leucine-rich repeat protein 18	1.6	$5x10^{-2}$
EFHD2	EF-hand domain family, member D2	1.5	$5x10^{-2}$
HIST1H2BK	histone cluster 1, H2bk	1.5	$5x10^{-2}$
KIF13B	kinesin family member 13B	1.4	$5x10^{-2}$
ZNF189	zinc finger protein 189	1.3	$5x10^{-2}$

Supplemental Table II. Genes Differently Expressed Between Nonsmoker-high MUC5AC Expressors Compared to Nonsmoker-low MUC5AC Expressors (cont., page 13)

Gene symbol[1]	Gene name	Fold-change[2]	p value[3]
GOLM1	golgi membrane protein 1	1.4	$5x10^{-2}$
BMS1	BMS1 homolog, ribosome assembly protein (yeast)	1.4	$5x10^{-2}$
MGA	MAX gene associated	1.5	$5x10^{-2}$
**STXBP6**	syntaxin binding protein 6 (amisyn)	1.6	$5x10^{-2}$
TARS	threonyl-tRNA synthetase	1.4	$5x10^{-2}$
CEACAM7	carcinoembryonic antigen-related cell adhesion molecule 7	1.6	$5x10^{-2}$
NMNAT1	nicotinamide nucleotide adenylyltransferase 1	1.5	$5x10^{-2}$
SNRPA1	small nuclear ribonucleoprotein polypeptide A'	1.4	$5x10^{-2}$
AHR	aryl hydrocarbon receptor	1.8	$5x10^{-2}$
CSNK1A1	casein kinase 1, alpha 1	1.2	$5x10^{-2}$
**XBP1**	X-box binding protein 1	1.4	$5x10^{-2}$
FZD1	frizzled homolog 1 (Drosophila)	1.3	$5x10^{-2}$
HSPA14	heat shock 70kDa protein 14	1.3	$5x10^{-2}$
SETD7	SET domain containing (lysine methyltransferase) 7	1.5	$5x10^{-2}$
ZBTB3	zinc finger and BTB domain containing 3	1.6	$5x10^{-2}$
C4orf19	chromosome 4 open reading frame 19	1.3	$5x10^{-2}$
BAIAP2L1	BAI1-associated protein 2-like 1	1.3	$5x10^{-2}$
BOD1	biorientation of chromosomes in cell division 1	1.2	$5x10^{-2}$
FBXW2	F-box and WD repeat domain containing 2	1.4	$5x10^{-2}$
GPATCH4	G patch domain containing 4	1.3	$5x10^{-2}$
LOC651250	hypothetical LOC651250	1.3	$5x10^{-2}$
LSG1	large subunit GTPase 1 homolog (S. cerevisiae)	1.5	$5x10^{-2}$
SLC45A4	solute carrier family 45, member 4	1.3	$5x10^{-2}$
SNRNP200	small nuclear ribonucleoprotein 200kDa (U5)	1.3	$5x10^{-2}$
TBL2	transducin (beta)-like 2	1.3	$5x10^{-2}$
TMPRSS2	transmembrane protease, serine 2	1.4	$5x10^{-2}$
TP53I3	tumor protein p53 inducible protein 3	1.5	$5x10^{-2}$
ZBTB7A	zinc finger and BTB domain containing 7A	1.4	$5x10^{-2}$
CDK6	cyclin-dependent kinase 6	1.5	$5x10^{-2}$
MAML1	mastermind-like 1 (Drosophila)	1.2	$5x10^{-2}$
DHRS3	dehydrogenase/reductase (SDR family) member 3	1.4	$5x10^{-2}$
EVI1	ecotropic viral integration site 1	1.3	$5x10^{-2}$
**Downregulated**			
RIMKLB	ribosomal modification protein rimK-like family member B	0.7	$4x10^{-2}$
CUGBP2	CUG triplet repeat, RNA binding protein 2	0.7	$3x10^{-2}$
FLJ40330	hypothetical LOC645784	0.7	$5x10^{-2}$
IGHG1	Immunoglobulin heavy constant gamma 1 (G1m marker)	0.6	$3x10^{-2}$
CYP24A1	cytochrome P450, family 24, subfamily A, polypeptide 1	0.6	$5x10^{-2}$
C5orf23	chromosome 5 open reading frame 23	0.6	$3x10^{-2}$
TFPI	tissue factor pathway inhibitor (lipoprotein-associated coagulation inhibitor)	0.6	$5x10^{-2}$
LOC441461	hypothetical LOC441461	0.5	$2x10^{-2}$
MYL4	myosin, light chain 4, alkali; atrial, embryonic	0.5	$4x10^{-2}$
SUSD2	sushi domain containing 2	0.5	$3x10^{-2}$
SLC25A37	solute carrier family 25, member 37	0.4	$2x10^{-2}$

Supplemental Table II. Genes Differently Expressed Between Nonsmoker-high MUC5AC Expressors Compared to Nonsmoker-low MUC5AC Expressors (cont., page 14)

Gene symbol[1]	Gene name	Fold-change[2]	p value[3]
ABI3BP	ABI family, member 3 (NESH) binding protein	0.4	$2x10^{-2}$
ENPP2	ectonucleotide pyrophosphatase/phosphodiesterase 2	0.4	$4x10^{-2}$
CAV1	caveolin 1, caveolae protein, 22kDa	0.2	$5x10^{-2}$
HBG1 /// HBG2	hemoglobin, gamma A /// hemoglobin, gamma G	0.2	$2x10^{-2}$

[1] The MUC5AC-core genes are highlighted with bold font.
[2] Fold-difference, nonsmoker-high MUC5AC expressors compared to nonsmoker-low MUC5AC expressors.
[3] p value, nonsmoker-high MUC5AC expressors compared nonsmoker- low MUC5AC expressors. Significant differences of gene expression between high MUC5AC expressors and low MUC5AC expressors were determined by an unequal variances Student's t test followed by Benjamini-Hochberg Correction ($p<0.05$).

Supplemental Table III. Function Annotation Term Enrichment Analysis of Differently Expressed Genes Between Nonsmoker-high MUC5AC Expressors Compared Nonsmoker-Low MUC5AC Expressors[1]

Term[2]	Count[3]	%[4]	p value[5]
Phosphoprotein	495	53.0	$6x10^{-22}$
Golgi apparatus	70	7.5	$9x10^{-10}$
Endoplasmic reticulum	69	7.4	$6x10^{-6}$
Alternative splicing	430	46.0	$5x10^{-5}$
Acetylation	177	19.0	$1x10^{-4}$
ER-golgi transport	17	1.8	$1x10^{-4}$
Protein transport	47	5.0	$5x10^{-4}$
Signal-anchor	40	4.3	$4x10^{-3}$
Unfolded protein response	8	0.9	$4x10^{-3}$
Transport	114	12.2	$4x10^{-3}$
Glycosyltransferase	25	2.7	$4x10^{-3}$
ATP-binding	93	10.0	$6x10^{-3}$

[1] The analysis were performed in The Database for Annotation, Visualization and Integrated Discovery (DAVID). Only upregulated genes were used.

[2] DAVID default SP_PIR_KEYWORDS.

[3] Number of differently expressed genes between high MUC5AC expressors and low MUC5AC expressors containing the term.

[4] Percentage of differentially expressed genes containing the term.

[5] p value after Benjanmini correction.

Supplemental Table IV. Effect of Smoking on Expression of MUC5AC-core Genes

Gene symbol	Gene name	Fold-difference, smokers to nonsmokers[1]	p value[2]
**Mucus components**			
TFF3	trefoil factor 3 (intestinal)	1.8	**$2x10^{-5}$**
TFF1	trefoil factor 1	2.0	**$6x10^{-5}$**
**Mucin producing cell differentiation-related transcription factors**			
SPDEF	SAM pointed domain containing ets transcription factor	1.4	**$5x10^{-4}$**
FOXA3	forkhead box A3	1.4	**$1x10^{-5}$**
SOX2	SRY (sex determining region Y)-box 2	-1.1	0.3
KLF4	Kruppel-like factor 4 (gut)	1.1	0.4
**Mucus-producing cell differentiation-related pathways or mediators**			
HES1	hairy and enhancer of split 1, (Drosophila)	-1.2	$6x10^{-2}$
TSTA3	tissue specific transplantation antigen P35B	1.0	0.8
LRRFIP2	leucine rich repeat (in FLII) interacting protein 2	1.1	**$2x10^{-3}$**
KRAS	v-Ki-ras2 Kirsten rat sarcoma viral oncogene homolog	1.1	0.3
MAPK13	mitogen-activated protein kinase 13	1.0	0.7
RPS6KA3	ribosomal protein S6 kinase, 90kDa, polypeptide 3	-1.1	0.3
CTSC	cathepsin C	1.2	**$7x10^{-3}$**
SERPINB4	serpin peptidase inhibitor, clade B (ovalbumin), member 4	-1.6	**$8x10^{-3}$**
PLA2G4A	phospholipase A2, group IVA (cytosolic, calcium-dependent)	1.2	**$4x10^{-2}$**
**Post-translational modification of mucins**			
AGR2	anterior gradient homolog 2 (Xenopus laevis)	1.6	**$2x10^{-5}$**
GNE	glucosamine (UDP-N-acetyl)-2-epimerase/N-acetylmannosamine kinase	1.1	0.4
GALNT4	UDP-N-acetyl-alpha-D-galactosamine:polypeptide N-acetylgalactosaminyltransferase 4 (GalNAc-T4)	-1.1	0.5
GALNT7	UDP-N-acetyl-alpha-D-galactosamine:polypeptide N-acetylgalactosaminyltransferase 7 (GalNAc-T7)	1.3	**$4x10^{-3}$**
GALNT12	UDP-N-acetyl-alpha-D-galactosamine:polypeptide N-acetylgalactosaminyltransferase 12 (GalNAc-T12)	1.3	**$3x10^{-4}$**
PDIA5	protein disulfide isomerase family A, member 5	1.1	**$4x10^{-2}$**
FUT3	fucosyltransferase 3 (galactoside 3(4)-L-fucosyltransferase, Lewis blood group)	1.5	**$2x10^{-5}$**
FUT6	fucosyltransferase 6 (alpha (1,3) fucosyltransferase)	1.2	**$5x10^{-4}$**
ST6GAL1	ST6 beta-galactosamide alpha-2,6-sialyltranferase 1	-1.2	**$4x10^{-2}$**
ST8SIA1	ST8 alpha-N-acetyl-neuraminide alpha-2,8-sialyltransferase 1	1.4	**$5x10^{-4}$**
CHST6	carbohydrate (N-acetylglucosamine 6-O) sulfotransferase 6	1.1	$8x10^{-2}$

Supplemental Table IV. Effect of Smoking on Expression of MUC5AC-core Genes (cont., page 2)

Gene symbol	Gene name	Fold-difference, smokers to nonsmokers[1]	p value[2]
GALNT5	UDP-N-acetyl-alpha-D-galactosamine:polypeptide N-acetylgalactosaminyltransferase 5 (GalNAc-T5)	1.6	**$9x10^{-8}$**
GALNT6	UDP-N-acetyl-alpha-D-galactosamine:polypeptide N-acetylgalactosaminyltransferase 6 (GalNAc-T6)	1.5	**$5x10^{-5}$**
**Vesicle transport**			
MIA3	melanoma inhibitory activity family, member 3	1.2	**$1x10^{-2}$**
SURF4	surfeit 4	1.0	0.7
KDELR2	KDEL (Lys-Asp-Glu-Leu) endoplasmic reticulum protein retention receptor 2	-1.0	0.6
KDELR3	KDEL (Lys-Asp-Glu-Leu) endoplasmic reticulum protein retention receptor 3	1.5	**$3x10^{-4}$**
ITSN1	intersectin 1 (SH3 domain protein)	1.0	0.7
ERGIC1	endoplasmic reticulum-golgi intermediate compartment (ERGIC) 1	1.0	0.8
CKAP4	cytoskeleton-associated protein 4	1.3	**$2x10^{-3}$**
GOSR1	golgi SNAP receptor complex member 1	-1.1	**$2x10^{-3}$**
SYNJ2BP	synaptojanin 2 binding protein	1.1	0.4
MPPE1	Metallophosphoesterase 1	-1.3	**$4x10^{-3}$**
SEC31A	SEC31 homolog A (S. cerevisiae)	-1.0	0.4
ARF4	ADP-ribosylation factor 4	-1.1	$9x10^{-2}$
VPS13D	vacuolar protein sorting 13 homolog D (S. cerevisiae)	1.6	**$6x10^{-11}$**
SEC22B	SEC22 vesicle trafficking protein homolog B (S. cerevisiae)	-1.0	0.5
TPD52	tumor protein D52	1.1	0.3
**Endoplasmic reticulum stress-associated**			
CREB3L1	cAMP responsive element binding protein 3-like 1	1.5	**$2x10^{-5}$**
EDEM3	ER degradation enhancer, mannosidase alpha-like 3	1.1	0.4
XBP1	X-box binding protein 1	-1.1	0.5
EIF2AK3	eukaryotic translation initiation factor 2-alpha kinase 3	1.1	0.2
**Secretory granule-associated**			
SYTL2	synaptotagmin-like 2	1.2	**$9x10^{-3}$**
RAB3D	RAB3D, member RAS oncogene family	-1.0	1.0
SCIN	scinderin	1.2	$7x10^{-2}$
STXBP6	syntaxin binding protein 6 (amisyn)	1.0	0.8
RAB27B	RAB27B, member RAS oncogene family	1.2	**$4x10^{-2}$**
SYTL4	synaptotagmin-like 4	1.2	**$4x10^{-2}$**
SYTL5	synaptotagmin-like 5	-1.0	0.9
GSN	gelsolin (amyloidosis, Finnish type)	1.1	0.6

Supplemental Table IV. Effect of Smoking on Expression of MUC5AC-core Genes (cont., page 3)

Gene symbol	Gene name	Fold-difference, smokers to nonsmokers[1]	p value[2]
RIMS1	regulating synaptic membrane exocytosis 1	-1.2	$5x10^{-2}$
CASK	calcium/calmodulin-dependent serine protein kinase (MAGUK family)	-1.1	$9x10^{-2}$
MYO5B	myosin VB	1.1	0.2
MYO5C	myosin VC	-1.1	0.4
PCLO	piccolo (presynaptic cytomatrix protein)	-1.1	0.3
PAM	peptidylglycine alpha-amidating monooxygenase	1.2	**$1x10^{-2}$**
ATP6V0A4	ATPase, H+ transporting, lysosomal V0 subunit a4	1.7	**$1x10^{-8}$**
KIF5B	kinesin family member 5B	-1.1	0.2
CDC42EP5	CDC42 effector protein (Rho GTPase binding) 5	1.6	**$5x10^{-5}$**
**Mucus secretion-related regulators**			
PRSS23	protease, serine, 23	-1.0	0.9
DGKA	diacylglycerol kinase, alpha 80kDa	1.2	**$3x10^{-2}$**
PRKCD	protein kinase C, delta	-1.1	0.5
ITPR3	inositol 1,4,5-triphosphate receptor, type 3	1.0	0.7
PLCE1	phospholipase C, epsilon 1	-1.3	**$4x10^{-3}$**
**Mucus hypersecretory-related ion channels**			
SLC12A2	solute carrier family 12 (sodium/potassium/chloride transporters), member 2	-1.0	1.0
CLCA2	chloride channel accessory 2	-1.4	**$5x10^{-3}$**
SCNN1A	sodium channel, nonvoltage-gated 1 alpha	1.1	0.1
GABRP	gamma-aminobutyric acid (GABA) A receptor, pi	-1.1	0.7

[1] Fold change, healthy smokers compared healthy nonsmokers.

[2] p value, healthy smokers compared healthy nonsmokers. Significant differences of gene expression between healthy smokers compared healthy nonsmokers were determined by an unequal variance Student's t test followed by Benjamini-Hochberg Correction (p <0.05, **bold font**).

Supplemental Table V. Literature Supported Links for Figure 6

Links shown in Figure 6	Note[1]	Ref
KLF4 and mucus producing cells	Colon of gene knock-out mice and conjunctiva of conditional gene knockout mice	[5]
SOX2 and mucus producing cells	Airway of conditional gene knockout mice	[4]
SPDEF and mucus producing cells	Airway of conditional gene knockout mice	[3]
Wnt pathway and mucus producing cells	Airway of conditional transgenic mice	[8]
Notch pathway and mucus producing cells	Airway of conditional transgenic mice	[39]
MAPK pathway and mucus producing cells	Inhibition *in vitro*	[10]
CREB3L1 affect KDELR3, SEC31A, HES1, XBP1 gene expression	Transfection in human cells	[21]
HES1 affect KLF4 gene expression	Transfection in human cells	[40]
XBP1 affect EDEM2, EIF2AK3 gene expression	Knockout murine cell	[41,42]
HES1 and TSTA3 belong to Notch pathway	General annotation	[20]
LRRFIP2 belongs to Wnt pathway	General annotation	[20]
MAPK13, KRAS, RPS6KA3 belong to MAPK pathway	General annotation	[20]
SPDEF affect AGR2, GALNT7, GALNT4, FOXA3 gene expression	Airway of conditional gene knockout mice	[3]
GNE affect ST8SIA1 gene expression	Transfection in human cells	[43]
AGR2 affect TFF3 gene expression	Intestine of gene knock-out mice	[15]
SOX2 affect AGR2 gene expression	Transfection in human cells	[4]

[1] Indicates the studies in the literature that were used to build the connection.

Supplemental Table VI. Effect of Asthma on MUC5AC-core Genes[1]

Gene symbol	Gene name	Fold-change[2]	p value[3]
**Up-regulated**			
PDIA5	protein disulfide isomerase family A, member 5	1.2	$3x10^{-3}$
GSN	gelsolin (amyloidosis, Finnish type)	1.4	$4x10^{-2}$
TFF3	trefoil factor 3 (intestinal)	1.3	$4x10^{-2}$
KLF4	Kruppel-like factor 4 (gut)	1.3	$4x10^{-2}$
SERPINB4	serpin peptidase inhibitor, clade B (ovalbumin), member 4	1.2	$4x10^{-2}$
KDELR3	KDEL (Lys-Asp-Glu-Leu) endoplasmic reticulum protein retention receptor 3	1.3	$4x10^{-2}$
**Down-regulated**			
RIMS1	regulating synaptic membrane exocytosis 1	0.8	$4x10^{-2}$
**No change**			
SURF4	surfeit 4	1.1	$5x10^{-2}$
SPDEF	SAM pointed domain containing ets transcription factor	1.2	$5x10^{-2}$
PCLO	piccolo (presynaptic cytomatrix protein)	0.8	$5x10^{-2}$
AGR2	anterior gradient homolog 2 (Xenopus laevis)	1.4	$7x10^{-2}$
SOX2	SRY (sex determining region Y)-box 2	1.1	$7x10^{-2}$
CDC42EP5	CDC42 effector protein (Rho GTPase binding) 5	1.7	$8x10^{-2}$
GALNT12	UDP-N-acetyl-alpha-D-galactosamine:polypeptide N-acetylgalactosaminyltransferase 12 (GalNAc-T12)	1.2	$8x10^{-2}$
FUT3	fucosyltransferase 3 (galactoside 3(4)-L-fucosyltransferase, Lewis blood group)	1.3	$9x10^{-2}$
DGKA	diacylglycerol kinase, alpha 80kDa	1.2	$9x10^{-2}$
TPD52	tumor protein D52	1.2	$9x10^{-2}$
TFF1	trefoil factor 1	1.3	0.2
SYTL2	synaptotagmin-like 2	1.1	0.2
GALNT4	UDP-N-acetyl-alpha-D-galactosamine:polypeptide N-acetylgalactosaminyltransferase 4 (GalNAc-T4)	1.3	0.2
KDELR2	KDEL (Lys-Asp-Glu-Leu) endoplasmic reticulum protein retention receptor 2	1.1	0.2
GOSR1	golgi SNAP receptor complex member 1	1.1	0.2
CREB3L1	cAMP responsive element binding protein 3-like 1	1.1	0.2
FUT6	fucosyltransferase 6 (alpha (1,3) fucosyltransferase)	1.1	0.2
ST6GAL1	ST6 beta-galactosamide alpha-2,6-sialyltranferase 1	1.1	0.2
KIF5B	kinesin family member 5B	1.1	0.2
GALNT7	UDP-N-acetyl-alpha-D-galactosamine:polypeptide N-acetylgalactosaminyltransferase 7 (GalNAc-T7)	1.1	0.2
EDEM3	ER degradation enhancer, mannosidase alpha-like 3	1.2	0.2
MIA3	melanoma inhibitory activity family, member 3	1.1	0.2
MYO5B	myosin VB	1.1	0.2
CKAP4	cytoskeleton-associated protein 4	1.1	0.3
FOXA3	forkhead box A3	1.1	0.3
MAPK13	mitogen-activated protein kinase 13	1.1	0.3

Supplemental Table VI. Effect of Asthma on MUC5AC-core Genes[1] (Cont., page 2)

Gene symbol	Gene name	Fold-change[2]	p value[3]
SEC22B	SEC22 vesicle trafficking protein homolog B (S. cerevisiae)	1.1	0.3
PLCE1	phospholipase C, epsilon 1	1.2	0.3
ITPR3	inositol 1,4,5-triphosphate receptor, type 3	1.2	0.3
PLA2G4A	phospholipase A2, group IVA (cytosolic, calcium-dependent)	1.1	0.3
XBP1	X-box binding protein 1	0.9	0.3
SYTL4	synaptotagmin-like 4	1.1	0.3
RAB3D	RAB3D, member RAS oncogene family	1.1	0.3
HES1	hairy and enhancer of split 1, (Drosophila)	1.0	0.3
GABRP	gamma-aminobutyric acid (GABA) A receptor, pi	1.1	0.4
RAB27B	RAB27B, member RAS oncogene family	1.1	0.4
SCIN	scinderin	1.1	0.4
RPS6KA3	ribosomal protein S6 kinase, 90kDa, polypeptide 3	1.1	0.4
MPPE1	Metallophosphoesterase 1	1.1	0.4
ARF4	ADP-ribosylation factor 4	1.1	0.4
TSTA3	tissue specific transplantation antigen P35B	1.0	0.4
ERGIC1	endoplasmic reticulum-golgi intermediate compartment (ERGIC) 1	1.1	0.5
CASK	calcium/calmodulin-dependent serine protein kinase (MAGUK family)	1.1	0.5
KRAS	v-Ki-ras2 Kirsten rat sarcoma viral oncogene homolog	1.0	0.5
PRKCD	protein kinase C, delta	1.0	0.5
VPS13D	vacuolar protein sorting 13 homolog D (S. cerevisiae)	1.0	0.5
SLC12A2	solute carrier family 12 (sodium/potassium/chloride transporters), member 2	0.9	0.5
ATP6V0A4	ATPase, H+ transporting, lysosomal V0 subunit a4	1.1	0.6
CHST6	carbohydrate (N-acetylglucosamine 6-O) sulfotransferase 6	1.0	0.6
ST8SIA1	ST8 alpha-N-acetyl-neuraminide alpha-2,8-sialyltransferase 1	1.0	0.6
EIF2AK3	eukaryotic translation initiation factor 2-alpha kinase 3	1.1	0.6
GALNT5	UDP-N-acetyl-alpha-D-galactosamine:polypeptide N-acetylgalactosaminyltransferase 5 (GalNAc-T5)	1.0	0.6
GALNT6	UDP-N-acetyl-alpha-D-galactosamine:polypeptide N-acetylgalactosaminyltransferase 6 (GalNAc-T6)	1.1	0.6
SYNJ2BP	synaptojanin 2 binding protein	1.0	0.7
CLCA2	chloride channel accessory 2	1.0	0.7
SEC31A	SEC31 homolog A (S. cerevisiae)	1.0	0.8
LRRFIP2	leucine rich repeat (in FLII) interacting protein 2	1.0	0.8
ITSN1	intersectin 1 (SH3 domain protein)	1.0	0.8
CTSC	cathepsin C	1.0	0.8
STXBP6	syntaxin binding protein 6 (amisyn)	1.0	0.8
SYTL5	synaptotagmin-like 5	1.0	0.9
GNE	glucosamine (UDP-N-acetyl)-2-epimerase/N-acetylmannosamine kinase	1.0	0.9
MYO5C	myosin VC	1.0	0.9
PRSS23	protease, serine, 23	1.0	0.9

Supplemental Table VI. Effect of Asthma on MUC5AC-core Genes[1] (Cont., page 3)

Gene symbol	Gene name	Fold-change[2]	p value[3]
PAM	peptidylglycine alpha-amidating monooxygenase	1.0	1
SCNN1A	sodium channel, nonvoltage-gated 1 alpha	1.0	1

[1] Expression data (GSE4302, asthma study [44]) of asthma at base line (n=42) and healthy nonsmokers (n=28) were used.

[2] Fold-change, asthma compared to healthy nonsmokers.

[3] p value, asthma compared to healthy nonsmokers. Significant differences of gene expression between asthma and healthy nonsmokers were determined by an unequal variances Student's t test followed by Benjamini-Hochberg Correction ($p<0.05$)

Supplementary Table VII. MUC5AC-core Genes are Enriched in MUC5AC-associated Transcriptome of Human Airway Epithelium in Asthma Study[1,2]

Gene symbol[3]	Spearman's rank correlation coefficient	p value
TFF3	0.7	$5x10^{-12}$
TFF1	0.7	$1x10^{-9}$
KDELR3	0.6	$3x10^{-7}$
PDIA5	0.5	$2x10^{-6}$
AGR2	0.5	$4x10^{-6}$
FUT3	0.5	$8x10^{-6}$
SPDEF	0.5	$1x10^{-5}$
TSTA3	0.5	$8x10^{-5}$
ERGIC1	0.5	$8x10^{-5}$
MAPK13	0.4	$1x10^{-4}$

[1] Expression data (GSE4302, asthma study [44]) of asthma at base line (n=42) and healthy control (n=28) were used.

[2] Genome-wide spearman's rank correlations to MUC5AC (214385_s_at) were calculated.

[3] Ten out of 73 MUC5AC-core genes are presented in the top 300 MUC5AC correlated genes from this asthma study, which is significant enriched ($p<10^{-4}$, chi-square test).

Supplementary Table VIII. Enriched Pathway Analysis for ERBB2 Correlated Genes[1]

Rank	Enriched pathway[2]	Number of ERBB2 correlated genes involved in the pathway	p value[3]
**PANTHER pathway analysis**			
1	Ubiquitin proteasome pathway	6	0.02
2	**EGF receptor signaling pathway**	7	0.06
3	PDGF signaling pathway	8	0.07
4	Integrin signalling pathway	9	0.07
5	JAK/STAT signaling pathway	3	0.07
**BIOCARTA pathway analysis**			
1	ADP-Ribosylation Factor	4	0.03
2	**Role of ERBB2 in Signal Transduction and Oncology**	4	0.05
3	Erk and PI-3 Kinase Are Necessary for Collagen Binding in Corneal Epithelia	4	0.06
4	CxCR4 Signaling Pathway	4	0.06
5	IL 4 signaling pathway	3	0.07

[1] Expression data was based on microarray data from 60 healthy nonsmokers and 72 healthy smokers. ERBB2 (210930_s_at) correlated genes with Pearson coefficient >0.7 were submitted to the Database for Annotation, Visualization and Integrated Discovery (DAVID) for pathway analysis.

[2] EGF receptors related pathways were highlighted.

[3] Modified Fisher Exact p value. The smaller, the more enriched.

References

1. Wiede A, Jagla W, Welte T, Kohnlein T, Busk H, Hoffmann W: **Localization of TFF3, a new mucus-associated peptide of the human respiratory tract.** *Am J Respir Crit Care Med* 1999, **159:**1330-1335.

2. Madsen J, Nielsen O, Tornoe I, Thim L, Holmskov U: **Tissue localization of human trefoil factors 1, 2, and 3.** *J Histochem Cytochem* 2007, **55:**505-513.

3. Chen G, Korfhagen TR, xu Y, Kitzmiller J, Wert SE, Maeda Y, Gregorieff A, Clevers H, Whitsett JA: **SPDEF is required for mouse pulmonary goblet cell differentiation and regulates a network of genes associated with mucus production.** *J Clin Invest* 2009, **119:**2914-2924.

4. Tompkins DH, Besnard V, Lange AW, Wert SE, Keiser AR, Smith AN, Lang R, Whitsett JA: **Sox2 is required for maintenance and differentiation of bronchiolar Clara, ciliated, and goblet cells.** *PLoS One* 2009, **4:**e8248.

5. Katz JP, Perreault N, Goldstein BG, Lee CS, Labosky PA, Yang VW, Kaestner KH: **The zinc-finger transcription factor Klf4 is required for terminal differentiation of goblet cells in the colon.** *Development* 2002, **129:**2619-2628.

6. Zheng x, Tsuchiya K, Okamoto R, Iwasaki M, Kano Y, Sakamoto N, Nakamura T, Watanabe M: **Suppression of hath1 gene expression directly regulated by hes1 via notch signaling is associated with goblet cell depletion in ulcerative colitis.** *Inflamm Bowel Dis* 2011, **17:**2251-2260.

7. Waterhouse CC, Johnson S, Phillipson M, Zbytnuik L, Petri B, Kelly M, Lowe JB, Kubes P: **Secretory cell hyperplasia and defects in Notch activity in a mouse model of leukocyte adhesion deficiency type II.** *Gastroenterology* 2010, **138:**1079-1090.

8. Mucenski ML, Nation JM, Thitoff AR, Besnard V, xu Y, Wert SE, Harada N, Taketo MM, Stahlman MT, Whitsett JA: **Beta-catenin regulates differentiation of respiratory epithelial cells in vivo.** *Am J Physiol Lung Cell Mol Physiol* 2005, **289:**L971-L979.

9. Marchetti A, Buttitta F, Pellegrini S, Chella A, Bertacca G, Filardo A, Tognoni V, Ferreli F, Signorini E, Angeletti CA et al.: **Bronchioloalveolar lung carcinomas: K-ras mutations are constant events in the mucinous subtype.** *J Pathol* 1996, **179:**254-259.

10. Fujisawa T, Ide K, Holtzman MJ, Suda T, Suzuki K, Kuroishi S, Chida K, Nakamura H: **Involvement of the p38 MAPK pathway in IL-13-induced mucous cell metaplasia in mouse tracheal epithelial cells.** *Respirology* 2008, **13:**191-202.

11. Kim SW, Hong JS, Ryu SH, Chung WC, Yoon JH, Koo JS: **Regulation of mucin gene expression by CREB via a nonclassical retinoic acid signaling pathway.** *Mol Cell Biol* 2007, **27:**6933-6947.

12. Foley JP, Bolognese BS, Long E, Salmon M, Podolin PL: **Ozone and staphylococcal enterotocin B exposure induces emphysema and mucus production that is dependent upon**

cathepsin C expression [abstract]. *Am J Respir Crit Care Med* 2009,

13. Sivaprasad U, Askew DJ, Ericksen MB, Gibson AM, Stier MT, Brandt EB, Bass SA, Daines MO, Chakir J, Stringer KF et al.: **A nonredundant role for mouse Serpinb3a in the induction of mucus production in asthma.** *J Allergy Clin Immunol* 2011, **127:**254-61, 261.

14. Dif F, Wu YZ, Burgel PR, Ollero M, Leduc D, Aarbiou J, Borot F, Garcia-Verdugo I, Martin C, Chignard M et al.: **Critical role of cytosolic phospholipase A2{alpha} in bronchial mucus hypersecretion in CFTR-deficient mice.** *Eur Respir J* 2010, **36:**1120-1130.

15. Park SW, Zhen G, Verhaeghe C, Nakagami Y, Nguyenvu LT, Barczak AJ, Killeen N, Erle DJ: **The protein disulfide isomerase AGR2 is essential for production of intestinal mucus.** *Proc Natl Acad Sci U S A* 2009, **106:**6950-6955.

16. Gagiannis D, Orthmann A, Danssmann I, Schwarzkopf M, Weidemann W, Horstkorte R: **Reduced sialylation status in UDP-N-acetylglucosamine-2-epimerase/N-acetylmannosamine kinase (GNE)-deficient mice.** *Glycoconj J* 2007, **24:**125-130.

17. Thornton DJ, Rousseau K, McGuckin MA: **Structure and function of the polymeric mucins in airways mucus.** *Annu Rev Physiol* 2008, **70:**459-486.

18. Nancarrow DJ, Clouston AD, Smithers BM, Gotley DC, Drew PA, Watson DI, Tyagi S, Hayward NK, Whiteman DC: **Whole genome expression array profiling highlights differences in mucosal defense genes in Barrett's esophagus and esophageal adenocarcinoma.** *PLoS One* 2011, **6:**e22513.

19. Lopez-Ferrer A, de BC, Barranco C, Garrido M, Isern J, Carlstedt I, Reis CA, Torrado J, Real Fx: **Role of fucosyltransferases in the association between apomucin and Lewis antigen expression in normal and malignant gastric epithelium.** *Gut* 2000, **47:**349-356.

20. Rebhan M, Chalifa-Caspi V, Prilusky J, Lancet D: **GeneCards: a novel functional genomics compendium with automated data mining and query reformulation support.** *Bioinformatics* 1998, **14:**656-664.

21. Fox RM, Hanlon CD, Andrew DJ: **The CrebA/Creb3-like transcription factors are major and direct regulators of secretory capacity.** *J Cell Biol* 2010, **191:**479-492.

22. Hirao K, Natsuka Y, Tamura T, Wada I, Morito D, Natsuka S, Romero P, Sleno B, Tremblay LO, Herscovics A et al.: **EDEM3, a soluble EDEM homolog, enhances glycoprotein endoplasmic reticulum-associated degradation and mannose trimming.** *J Biol Chem* 2006, **281:**9650-9658.

23. Lee AH, Iwakoshi NN, Glimcher LH: **xBP-1 regulates a subset of endoplasmic reticulum resident chaperone genes in the unfolded protein response.** *Mol Cell Biol* 2003, **23:**7448-7459.

24. Yamaguchi Y, Larkin D, Lara-Lemus R, Ramos-Castaneda J, Liu M, Arvan P: **Endoplasmic reticulum (ER) chaperone regulation and survival of cells compensating for deficiency in the ER stress response kinase, PERK.** *J Biol Chem* 2008, **283:**17020-17029.

25. Saegusa C, Tanaka T, Tani S, Itohara S, Mikoshiba K, Fukuda M: **Decreased basal mucus secretion by Slp2-a-deficient gastric surface mucous cells.** *Genes Cells* 2006, **11:**623-631.

26. Davis CW, Dickey BF: **Regulated airway goblet cell mucin secretion.** *Annu Rev Physiol* 2008, **70:**487-512.

27. Ehre C, Rossi AH, Abdullah LH, De PK, Hill S, Olsen JC, Davis CW: **Barrier role of actin filaments in regulated mucin secretion from airway goblet cells.** *Am J Physiol Cell Physiol* 2005, **288:**C46-C56.

28. Castermans D, Volders K, Crepel A, Backx L, De VR, Freson K, Meulemans S, Vermeesch JR, Schrander-Stumpel CT, De RP et al.: **SCAMP5, NBEA and AMISYN: three candidate genes for autism involved in secretion of large dense-core vesicles.** *Hum Mol Genet* 2010, **19:**1368-1378.

29. Tomas A, Yermen B, Min L, Pessin JE, Halban PA: **Regulation of pancreatic beta-cell insulin secretion by actin cytoskeleton remodelling: role of gelsolin and cooperation with the MAPK signalling pathway.** *J Cell Sci* 2006, **119:**2156-2167.

30. Kogel T, Gerdes HH: **Maturation of secretory granules.** *Results Probl Cell Differ* 2010, **50:**1-20.

31. Varadi A, Ainscow EK, Allan VJ, Rutter GA: **Involvement of conventional kinesin in glucose-stimulated secretory granule movements and exocytosis in clonal pancreatic beta-cells.** *J Cell Sci* 2002, **115:**4177-4189.

32. Zhen G, Park SW, Nguyenvu LT, Rodriguez MW, Barbeau R, Paquet AC, Erle DJ: **IL-13 and epidermal growth factor receptor have critical but distinct roles in epithelial cell mucin production.** *Am J Respir Cell Mol Biol* 2007, **36:**244-253.

33. Niles RM, Christensen TG, Breuer R, Stone PJ, Snider GL: **Serine proteases stimulate mucous glycoprotein release from hamster tracheal ring organ culture.** *J Lab Clin Med* 1986, **108:**489-497.

34. Fahy JV, Dickey BF: **Airway mucus function and dysfunction.** *N Engl J Med* 2010, **363:**2233-2247.

35. Dolganov GM, Woodruff PG, Novikov AA, Zhang Y, Ferrando RE, Szubin R, Fahy JV: **A novel method of gene transcript profiling in airway biopsy homogenates reveals increased expression of a Na+-K+-Cl- cotransporter (NKCC1) in asthmatic subjects.** *Genome Res* 2001, **11:**1473-1483.

36. Loewen ME, Forsyth GW: **Structure and function of CLCA proteins.** *Physiol Rev* 2005, **85:**1061-1092.

37. Mall M, Grubb BR, Harkema JR, O'Neal WK, Boucher RC: **Increased airway epithelial Na+ absorption produces cystic fibrosis-like lung disease in mice.** *Nat Med* 2004, **10:**487-493.

38. xiang YY, Wang S, Liu M, Hirota JA, Li J, Ju W, Fan Y, Kelly MM, Ye B, Orser B et al.: **A GABAergic system in airway epithelium is essential for mucus overproduction in asthma.** *Nat Med* 2007, **13:**862-867.

39. Guseh JS, Bores SA, Stanger BZ, Zhou Q, Anderson WJ, Melton DA, Rajagopal J: **Notch signaling promotes airway mucous metaplasia and inhibits alveolar development.** *Development* 2009, **136:**1751-1759.

40. Ghaleb AM, Aggarwal G, Bialkowska AB, Nandan MO, Yang VW: **Notch inhibits expression of the Kruppel-like factor 4 tumor suppressor in the intestinal epithelium.** *Mol Cancer Res* 2008, **6:**1920-1927.

41. Olivari S, Galli C, Alanen H, Ruddock L, Molinari M: **A novel stress-induced EDEM variant regulating endoplasmic reticulum-associated glycoprotein degradation.** *J Biol Chem* 2005, **280:**2424-2428.

42. Sriburi R, Bommiasamy H, Buldak GL, Robbins GR, Frank M, Jackowski S, Brewer JW: **Coordinate regulation of phospholipid biosynthesis and secretory pathway gene expression in xBP-1(S)-induced endoplasmic reticulum biogenesis.** *J Biol Chem* 2007, **282:**7024-7034.

43. Wang Z, Sun Z, Li AV, Yarema KJ: **Roles for UDP-GlcNAc 2-epimerase/ManNAc 6-kinase outside of sialic acid biosynthesis: modulation of sialyltransferase and BiP expression, GM3 and GD3 biosynthesis, proliferation, and apoptosis, and ERK1/2 phosphorylation.** *J Biol Chem* 2006, **281:**27016-27028.

44. Woodruff PG, Boushey HA, Dolganov GM, Barker CS, Yang YH, Donnelly S, Ellwanger A, Sidhu SS, Dao-Pick TP, Pantoja C et al.: **Genome-wide profiling identifies epithelial cell genes associated with asthma and with treatment response to corticosteroids.** *Proc Natl Acad Sci U S A* 2007, **104:**15858-15863.

Supplemental Figure Legends

Supplemental Figure 1. Coordinated molecular events associated with mucus production identified by the "MUC5AC-core gene" list. In response to various stimulation, transcription factors of the mucin-producing cells are activated, with consequent increased expression of genes related to mucin biosynthesis, secreted protein quality control (ER stress related unfolded protein response and protein degradation), vesicle transportation, mucus secretion and mucus-related ion/water channels. Examples of "MUC5AC-core genes" in each of 9 categories described in the text are listed; for the complete list, see Table III and Supplemental Table I. More details about "MUC5AC-core genes" in mucus secretion are in Supplementary Figure 2.

Supplemental Figure 2. Coordinated molecular events associated with secretion-related events identified by the "MUC5AC-core gene" list. The concept of this figure is based on recent reviews [1, 2], which summarized mucus secretion related events deduced mainly from studies of non-airway epithelial cells or non-mucus-producing cells. To reach the apical cytoplasm membrane, MUC5AC containing granule moves from the microtube to actin filament with the help of RAB3/27, myosin II/V, granunophilin. This is followed by actin filament disruption and remodeling, which is regulated by scinderin, gelsolin and MARCKS. MARCKS is activated by protein kinase C (PKC). Further tethering, docking and priming of MUC5AC granule involved the interaction of multiple proteins, including RABs, RAB effectors, Munc13, Munc18, Syntaxin, Snap23/25, VAMP and Synatagmin. The secretion processes are regulated by intracellular second messengers, IP3 and DAG, which are generated by phospholipase C (PLC). IP3 binds to its receptor ITPR3 on endoplasmic reticulum to release Ca^{2+}. The secretion related MUC5AC core genes are marked as red on the figure according to their function. Some secretion-related MUC5AC-core genes are not shown in this figure, including PCLO, CASK, DKGA, PAM, ATP6V0A4, KIF5B and CDC42EP5. Abbreviation: MARCKS, myristoylated alanine-rich C ki-

nase substrate; PKC, protein kinase C; PLC, phospholipase C; DAG, diacylglycerol; IP3, inositol trisphosphate.

References

1. Davis CW, Dickey BF (2008) Regulated airway goblet cell mucin secretion. Annu Rev Physiol 70: 487-512.
2. Fahy JV, Dickey BF (2010) Airway mucus function and dysfunction. N Engl J Med 363: 2233-2247.

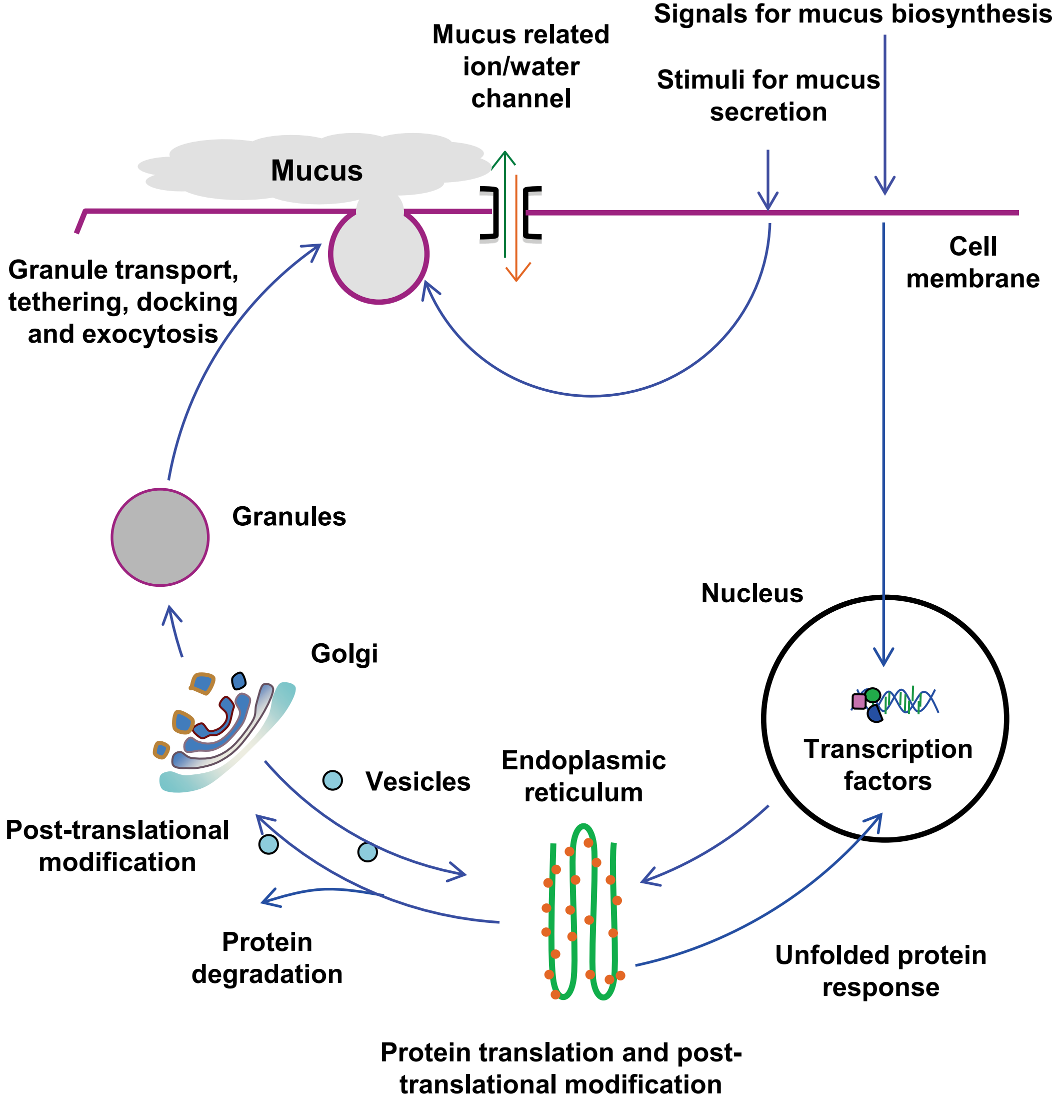

Mucus components
- TFF1, TFF3

Mucus-producing cell differentiation-related transcription factors
- SPDEF, FOXA3, KLF4

Mucus-producing cell differentiation-related pathways or mediators
- TSTA3, KRAS, SERPINB4

Post-translational modification of mucin
- AGR2, GALNT4, GALNT7

ER stress-related
- EDEM3, EIF2AK3, CREB3L1

Vesicle transport
- MIA3, KDELR3, ERGIC1

Secretory granule-associated
- SCIN, RAB3D (see supplementary figure 2 for detail)

Mucus secretion-related regulators
- PLCE1, ITPR3 (see supplemental Figure 2 for details)

Mucus hypersecretory-related ion channels
- SLC12A2

Supplementary Figure 1

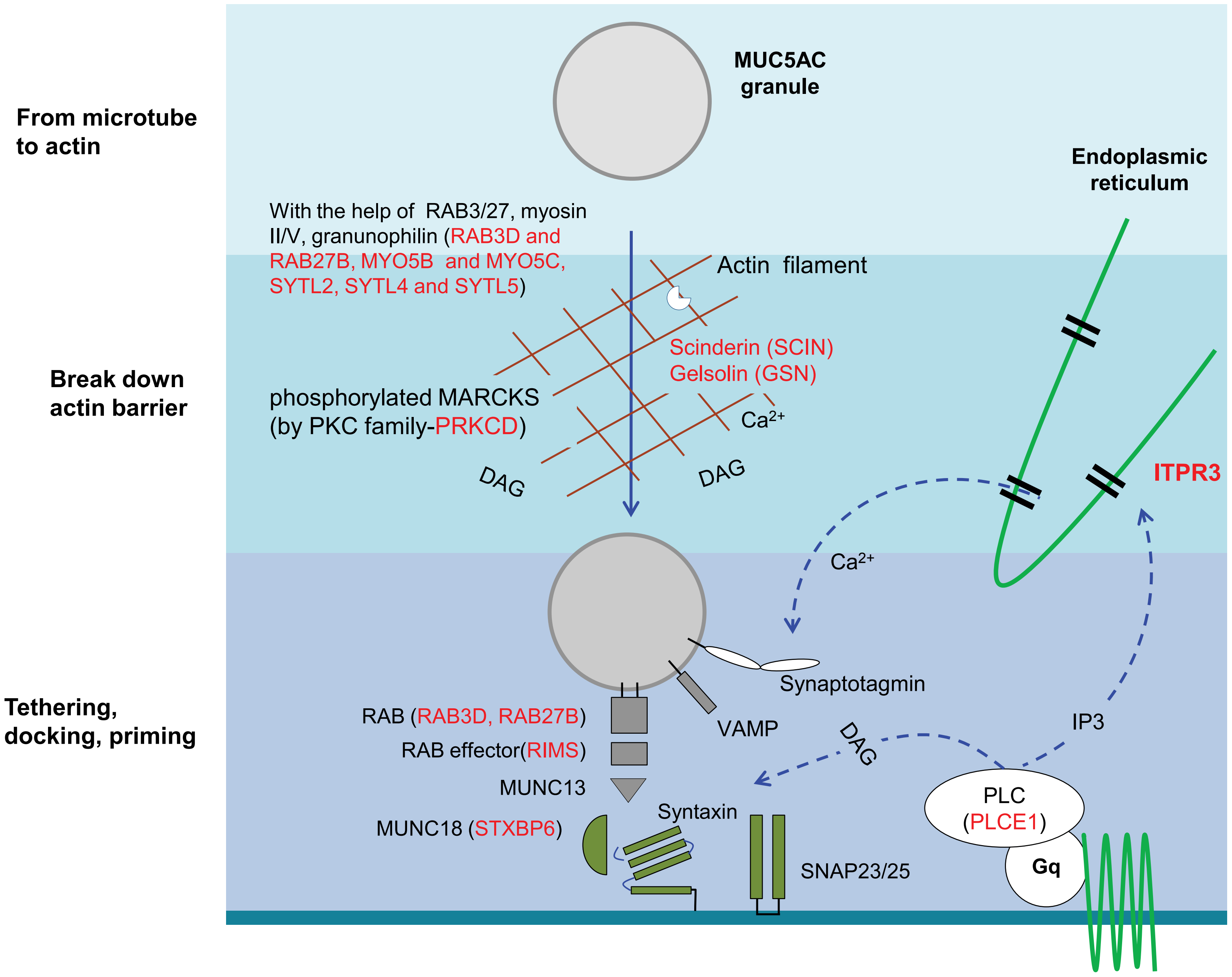

Supplemental Figure 2
